# Distinct mechanisms control genome recognition by p53 at its target genes linked to different cell fates

**DOI:** 10.1038/s41467-020-20783-z

**Published:** 2021-01-20

**Authors:** Marina Farkas, Hideharu Hashimoto, Yingtao Bi, Ramana V. Davuluri, Lois Resnick-Silverman, James J. Manfredi, Erik W. Debler, Steven B. McMahon

**Affiliations:** 1 Department of Biochemistry and Molecular Biology, Thomas Jefferson University, Philadelphia, PA USA; 2 Northwestern University Feinberg School of Medicine, Chicago, IL USA; 3 Icahn School of Medicine at Mount Sinai, New York, NY USA

**Keywords:** Next-generation sequencing, Tumour-suppressor proteins, Transcriptional regulatory elements, Molecular modelling

## Abstract

The tumor suppressor p53 integrates stress response pathways by selectively engaging one of several potential transcriptomes, thereby triggering cell fate decisions (e.g., cell cycle arrest, apoptosis). Foundational to this process is the binding of tetrameric p53 to 20-bp response elements (REs) in the genome (RRRCWWGYYYN_0-13_RRRCWWGYYY). In general, REs at cell cycle arrest targets (e.g. *p21*) are of higher affinity than those at apoptosis targets (e.g., *BAX*). However, the RE sequence code underlying selectivity remains undeciphered. Here, we identify molecular mechanisms mediating p53 binding to high- and low-affinity REs by showing that key determinants of the code are embedded in the DNA shape. We further demonstrate that differences in minor/major groove widths, encoded by G/C or A/T bp content at positions 3, 8, 13, and 18 in the RE, determine distinct p53 DNA-binding modes by inducing different Arg248 and Lys120 conformations and interactions. The predictive capacity of this code was confirmed in vivo using genome editing at the BAX RE to interconvert the DNA-binding modes, transcription pattern, and cell fate outcome.

## Abstract (web summary)

The tumor suppressor p53 is a master regulator of cellular stress response pathways, including cell cycle arrest and apoptosis. Here, the authors identify molecular mechanisms of p53 binding to high- and low-affinity p53 response elements in the genome, linked to cell cycle arrest and pro-apoptotic genes, respectively.

## Introduction

In response to different forms of stress, the tumor suppressor p53 selectively induces one of a variety of distinct transcriptional programs, each linked to a unique biological outcome (e.g., cell cycle arrest, apoptosis, DNA repair, ferroptosis)^1,2^. The central event in this process is the binding of tetrameric p53 to regulatory DNA motifs in the genome (i.e., response elements) of the consensus sequence RRRCWWGYYYN_0–13_RRRCWWGYYY (R = A or G, W = A or T, Y = C or T, N = any base)^3,4^. Focusing on this initial step, the prevailing model holds that p53 exhibits dose-dependent target gene activation, with REs linked to cell cycle arrest targets exhibiting higher affinity and REs linked to pro-apoptotic targets exhibiting lower affinity^5–7^. This has provided a conceptually satisfying model in which cells with modest DNA damage induce low levels of p53 that are capable of binding only at the high-affinity REs, thus providing the cell with the opportunity to repair its genome prior to advancing through the cell cycle. However, if levels of DNA damage exceed the cell’s repair capacity, sufficiently high quantities of p53 are induced and even the low-affinity REs linked to pro-apoptotic target genes are occupied and activated.

It has been previously proposed that the number of mismatched bps within a given RE determines its binding affinity^8,9^. However, REs with equal numbers of mismatches still show different affinities for p53, suggesting that mechanisms beyond the degree of a mismatch from the consensus are responsible. This is of physiological relevance because only 10% of 2183 in vivo verified p53 REs match the consensus sequence across all 20 bps^10^. p53 REs exhibit the highest sequence variability at the triplets RRR/YYY, which flank the core (CWWG) sequence^11^, and p53 tetramers interact with REs at those bases via Arg248 from the DNA minor groove and Lys120 from the DNA major groove^12^. Of note, different crystal structures and molecular dynamics simulations of p53 bound to DNA reveal that the Arg248 and especially Lys120 side chains are highly dynamic and can adopt multiple conformations when in complex with DNA^12–15^. This set of observations triggered our hypothesis that the interplay of Lys120 and Arg248 residues with variable sequences of REs might be a potential source of p53 selectivity.

The role of Lys120 in regulating p53 target gene selectivity has been previously explored. Acetylation of this residue positively correlates with the increase in the expression of pro-apoptotic genes^16,17^, while a conservative, cancer-derived mutation of Lys120 to Arg (K120R) selectively impairs the induction of apoptosis and binding of p53 to pro-apoptotic REs without impacting induction of cell cycle arrest^18^. However, the molecular mechanism responsible for p53 dose-dependent and Lys120-dependent target gene selectivity has not been identified. In our study, we describe a mechanism that explains the differential binding of p53 to two major sets of genomic targets—high-affinity and low-affinity REs, linked to cell cycle arrest and pro-apoptotic genes, respectively. Specifically, we show that RE minor groove width, which is encoded by G/C or A/T bp content at positions 3, 8, 13, and 18 in the RE and sensed by Arg248, determines two distinct p53 DNA-binding modes. Our findings advance a p53 RE code that mechanistically links RE sequence, DNA shape, DNA-binding mode, and biological output.

## Results

### An RE code distinguishes p53 effector pathways

**Fig. 1:**
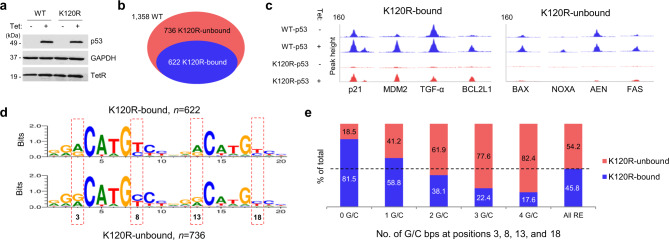
Differential recognition by p53 of distinct classes of REs is encoded in the G/C vs. A/T bp content at positions 3, 8, 13, and 18 in the RE sequence. **a** Representative western blot from three independent experiments showing the expression of WT and K120R-p53 in Tet-inducible H1299 cells. **b** Venn diagram depicting the number of significantly enriched (with at least 0.3 counts per million [cpm] reads) and previously identified^10^ WT p53 peaks, which are either bound (blue) or unbound (salmon) by K120R-p53 variant. **c** UCSC Genome Browser view of WT and K120R-p53 occupancy at selected K120R-bound and K120R-unbound-binding sites. Untreated (−) and Tet-treated (+) tracks for WT and K120R-p53 are shown in blue and salmon, respectively. **d** Motif analysis of K120R-bound (upper) and K120R-unbound (lower)-binding sites. Red dashed boxes highlight the differences at positions 3, 8, 13, and 18. **e** Percentage of either K120R-bound (blue) or K120R-unbound (salmon) REs with 0 (*n* = 178), 1 (*n* = 374), 2 (*n* = 501), 3 (*n* = 254), or 4 (*n* = 51) G/C bps at positions 3, 8, 13, and 18. The dashed line presents the percentage among all WT p53-binding sites (*n* = 1358), highlighting an increase in K120R sensitivity with an increase in G/C bp content. Tet tetracycline. TetR tetracycline repressor. Source data are provided as a Source Data file.

To gain insight into the role of Lys120 integrity in the recognition of specific RE sequences, we performed a chromatin-immunoprecipitation experiment and analyzed genome-wide occupancy of conditionally-expressed wild-type (WT) and K120R forms of p53 in the isogenic human H1299 lung cancer cell line that is null for endogenous p53 (Fig. 1a). In our ChIP-seq data we identified 1358 unique genomic-binding sites for WT p53 that were associated with previously identified, 20-bp REs (Fig. 1b)^10^. Of these 1358 REs, only 622 (45.8%) retained p53 binding when Lys120 was converted to arginine (termed “K120R-bound”). The remaining 736 (54.2%) sites lost p53 binding upon conversion of Lys120 to arginine (termed “K120R-unbound”) and exhibited generally lower WT p53 occupancy (Supplementary Fig. 1a, b). In agreement with previous biological observations^18^, the vast majority of REs at genes linked to the positive regulation of apoptosis were K120R-unbound in our studies. In contrast, REs at genes crucial for activation of processes such as cell cycle arrest or DNA repair were still bound by the K120R p53 variant (Fig. 1c and Supplementary Fig. 1c).

Further analysis of the nucleotide sequence of REs present within our ChIP-seq peaks revealed that the generic number of mismatches from the consensus RE did not directly correlate with in vivo occupancy by WT p53 or the dependence on Lys120 integrity (Supplementary Fig. 2). We therefore postulated that sequence identity at specific positions within the RE contributes to these two, presumably linked characteristics. To interrogate this hypothesis, consensus sequences were generated for the K120R-bound and K120R-unbound REs in our datasets. This analysis demonstrated that the major difference in the sequence of these two groups of REs resides at positions adjacent to the core (CWWG) sequence (Fig. 1d). More specifically, it demonstrated a preponderance of A/T bps at positions 3, 8, 13, and 18 in the K120R-bound group and G/C bps in the K120R-unbound group of REs. Additional analysis of the nucleotide content specifically at those positions confirmed this observation by showing that ~80% of the sites enriched with G/C bps were deficient in K120R-p53 binding (Fig. 1e).

**Fig. 2:**
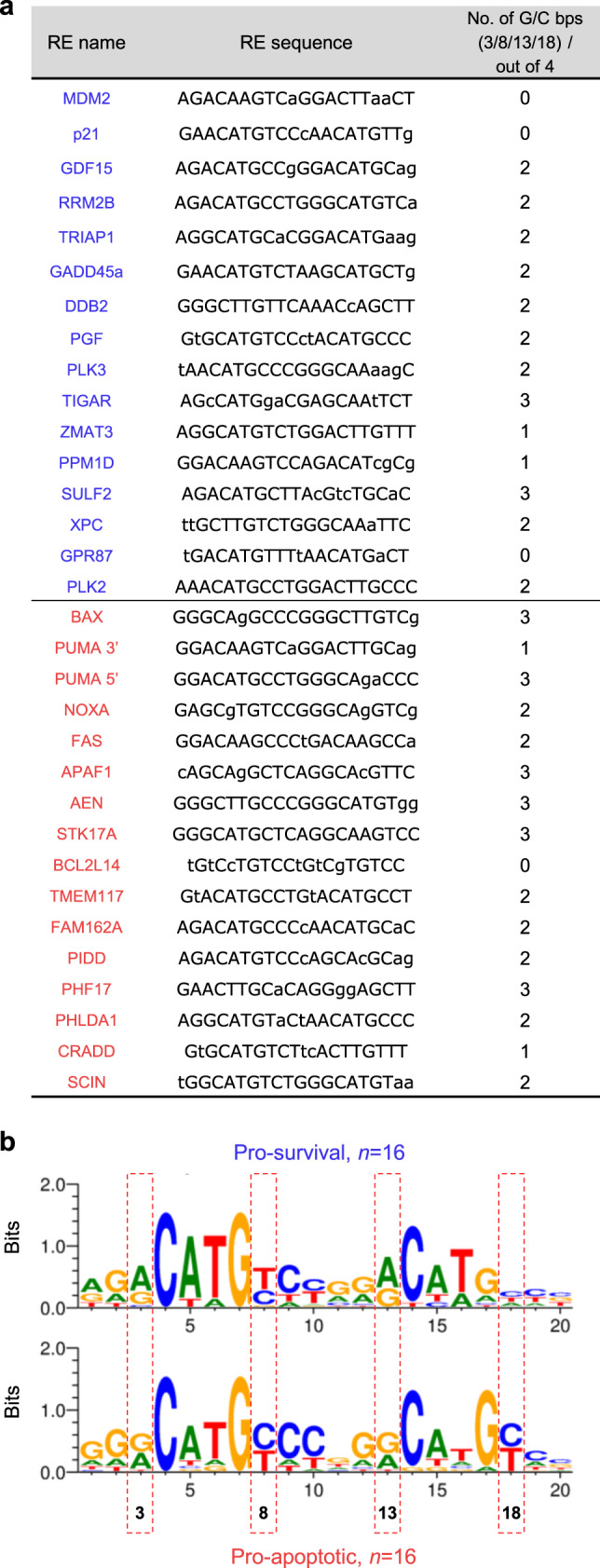
G/C vs. A/T bp content at positions 3, 8, 13, and 18 distinguishes REs linked to distinct p53-driven cell fates. **a** The list of sixteen pro-apoptotic p53 REs (bottom) and 16 previously confirmed REs corresponding to genes driving p53-dependent pro-survival response (top)^19^, with their nucleotide sequence. Lower-case letters in RE sequences indicate mismatches from p53 RE consensus. The last column shows the number of G/C bps at positions 3, 8, 13, and 18, emphasizing a tendency of pro-apoptotic REs having ≥ 2, and pro-survival REs having ≤ 2. **b** Motif analysis of pro-survival (top) and pro-apoptotic (bottom) REs listed in **a**. Positional analysis confirms an increased frequency of G/C bps at the core sequence-adjacent positions among pro-apoptotic REs and A/T bps among pro-survival REs. Source data are provided as a Source Data file.

As the role of Lys120 integrity in distinguishing between REs is linked to distinct cell fates^18^, we assessed whether the G/C vs. A/T bp content at positions 3, 8, 13, and 18 could similarly distinguish targets linked to distinct cell fates. We analyzed REs from our ChIP-seq data, which were linked to the genes previously identified as high-confidence p53 targets through the comprehensive meta-analysis of 319 previously published gene studies^19^. Remarkably, analysis of the REs connected to 16 pro-apoptotic targets revealed enrichment of G/C bps at these positions, similar to the sequence developed in Fig. 1d for the entire K120R-unbound group of REs (Fig. 2a,  b, bottom). An analysis of REs of sixteen, high-confidence, pro-survival targets revealed a consensus sequence with predominantly A/T bps at these positions, similar to the sequence developed in Fig. 1d for all K120R-bound REs (Fig. 2a, b, top). Additionally, REs linked to pro-apoptotic targets averaged more mismatches at the core (CWWG) sequence, relative to pro-survival REs. However, further testing showed that these mismatches only marginally decrease the p53 occupancy in vivo (Supplementary Fig. 3a), and do not regulate K120-dependency (Supplementary Fig. 3b). Interestingly, the G/C vs. A/T bp content at positions directly adjacent to the CWWG core still distinguished K120R-bound and K120R-unbound REs, independent of the status of the CWWG sequence itself (Supplementary Fig. 3c). Collectively, these findings suggest that differential recognition by p53 of distinct classes of REs is encoded, at least in part, by the A/T vs. G/C bp content at positions 3, 8, 13, and 18 within the primary DNA sequence.

### p53 uses two distinct RE-binding modes in vitro

To quantify p53 binding to K120R-bound and K120R-unbound REs in vitro, we measured the dissociation constants (*K*_d_) of natural and artificial REs, employing fluorescence polarization at 275 mM NaCl, which would best reflect in vivo conditions and which rendered non-specific DNA binding undetectable (Table 1 and Supplementary Fig. 4)^14,20,21^. For these experiments, we used a thermostable recombinant p53 protein lacking the unstructured N-terminal transactivation and proline-rich regions. This ΔN-p53 construct (residues 94-393) has previously been demonstrated to retain the same DNA-binding specificity as a full-length p53 protein^14,22^. Under the salt conditions described above, WT p53 exhibited high affinity for K120R-bound REs (23–97 nM), but one order of magnitude lower affinity for K120R-unbound REs (254–783 nM). This supports previous reports of higher affinity for cell cycle arrest REs such as p21 RE and lower affinity for pro-apoptotic REs such as BAX RE^6,21^. Conversion of Lys120 to Arg had relatively little impact on binding affinity for K120R-bound REs (43–80 nM), while significantly reducing binding affinity for K120R-unbound REs (800 nM to non-detectable). Together, these in vitro data recapitulate the K120R binding selectivity observed in vivo by ChIP-seq (Fig. 1b), and elsewhere^18^.

**Table 1:** Measured *K*_d_ values (in nM) of representative natural and artificial K120R-bound and K120R-unbound REs for WT and K120R-p53.

RE name	RE sequence	*K*_d_ [nM]			
		WT		K120R	
		200 mM NaCl	275 mM NaCl	200 mM NaCl	275 mM NaCl
p21	GAACATGTCCcAACATGTTg	26	23	35	43
MDM2	AGACAAGTCaGGACTTaaCT	20	97	36	72
APBB2	AGGCATGTCCcAACATGCCC	10	40	37	80
3Q05 pdb	GGGCATGTCTGGGCATGTCT	6	33	33	78
BAX	GGGCAgGCCCGGGCTTGTCg	22	460	280	N/A
NOXA	GAGCgTGTCCGGGCAgGTCg	45	446	68	∼2900
“Pro-apoptotic”	GGGCATGCCCGGGCATGCCC	15	254	23	∼3500
6FJ5 pdb	AGGCATGCCTAGGCATGCCT	8	783	140	800
BAX_8/13	GGGCAgGTCCGGACTTGTCg	8	40	94	168
NOXA_3/13	GAACgTGTCCGGACAgGTCg	23	60	14	181
p21_8/13	GAACATGCCCcAGCATGTTg	33	99	55	479
BAX_8/13 I/C	GGGCAgG**(I-)C**CCGG**I(-C)**CTTGTCg	9	83	150	N/A
6FJ5_8/18 I/C	AGGCATG**(I-)C**CTAGGCATG**(I-)C**CT	8	58	72	691

Lower-case letters in RE sequences indicate mismatches from p53 RE consensus. I (inosine) and C (cytosine) in bold indicate I/C bps. Letters in parenthesis indicate a nucleotide of the I-C bp found on the complementary strand. Underlined are core (CWWG) sequences.

*N/A* not available.

To gain further insight into the nature of K120R-bound and K120R-unbound-binding modes, we determined the *K*_d_ values at a lower salt concentration (200 mM NaCl). Under this condition, binding affinity for K120R-unbound REs was increased, when compared to the higher salt concentration, for both WT p53 (8–45 nM) and the K120R mutant (23–280 nM), suggesting an electrostatically-driven interaction. By contrast, recognition of K120R-bound REs increased only slightly at lower ionic strength for both WT (6–26 nM) and K120R (33–37 nM) proteins, consistent with predominantly non-polar interactions. We also analyzed an ideal, G/C-rich, presumably K120R-unbound RE termed “Pro-apoptotic RE” because the natural REs that we used in our binding assay (BAX RE and NOXA RE) contained mismatches with respect to the p53 consensus sequence, including the core (CWWG) sequence (indicated by lower-case letters in Table 1). The ideal RE confirmed the observed characteristics of the natural K120R-unbound REs, indicating that the mismatches had no effect on the binding mode or K120R sensitivity. Altogether, these in vitro binding data suggest that p53 employs a high-affinity, non-electrostatic effective binding mode for K120R-bound REs, while utilizing a lower affinity, electrostatic effective binding mode for K120R-unbound REs.

### p53 uses structurally distinct DNA-binding modes for different classes of REs

**Fig. 3:**
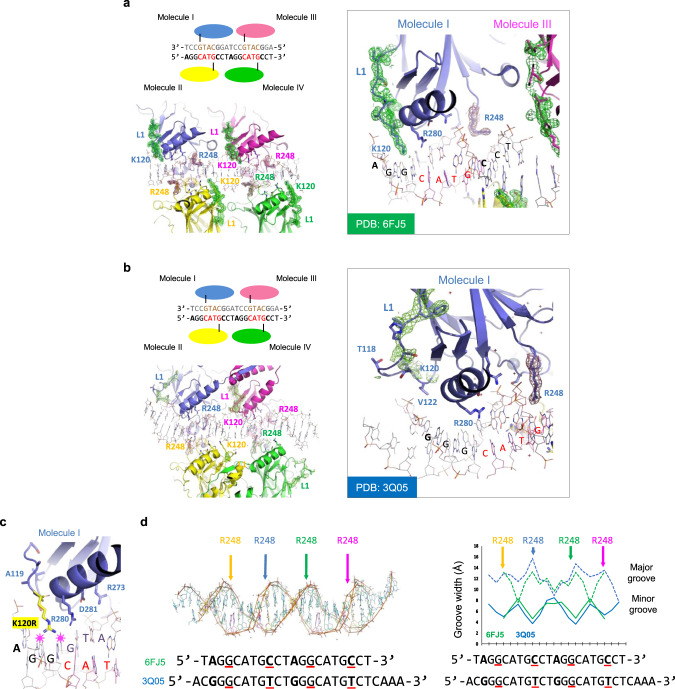
Structural analysis of p53 in complex with distinct REs. **a**, **b** Binding modes of p53 in complex with low-affinity, K120R-unbound (**a**) and high-affinity, K120R-bound RE (**b**). Top left: schematic representation of tetrameric p53 and DNA interactions. The CWWG core motifs are highlighted in red or gold. The four Arg280 residues of tetrameric p53 (molecules I to IV) that interact with guanine of the CWWG core regions are marked as black lines. DNA sequence differences between 6FJ5^23^ (**a**) and 3Q05^14^ (**b**) are shown in bold (positions 1, 8, 11, and 18). Lower left: overview of the p53 structure in complex with the respective RE. The 2F_o_–F_c_ electron density map contoured at 1σ above the mean is shown for the L1 loops in green and for the Arg248 residues in brown. Right: detailed view of molecule I-DNA interactions. **c** Modeling the Lys120 to Arg (K120R) mutation in the binding mode of low-affinity, K120R-unbound REs suggests steric clashes, indicated by pink stars with two guanine bases at positions 2 and 3. **d** Left: superimposition of the DNAs of 6FJ5 (green) and 3Q05 (blue). Arrows indicate significant differences between the two DNA structures and the approximate positions of the Arg248 residues colored as in **a** and **b** sensing the minor groove width. The aligned sequences are shown on the bottom, with differences in the 20-bp regions highlighted in bold, and positions 3, 8, 13, and 18 underlined in red. Right: width of major (dashed) and minor grooves (solid) calculated by the program 3DNA^47,48^. The plotted points correspond to positions between bps. Source data are provided as a Source Data file.

To investigate whether the two distinct p53-binding modes observed in vitro have correlates on the atomic level, we analyzed p53-DNA crystal structures available in the Protein Data Bank (PDB) and classified them into two groups based on Lys120 being ordered or disordered, as judged by an omit electron density map (Supplementary Table 1). In the first group, represented by a p53 structure complexed with a BAX-like RE (PDB: 6FJ5^23^, Fig. 3a and Supplementary Fig. 5), Lys120 protrudes into the major groove in an extended conformation and forms direct hydrogen bonds with the purine bases R12 and R13. On the opposite side of the double helix (minor groove), Arg248 adopts a bent conformation in which its guanidinium group engages in a direct or water-mediated electrostatic interaction with the phosphate(s) of Y8 and/or Y9, which base-pair with R12 and R13 (Fig. 3a and Supplementary Fig. 5). In the second group represented by a p53 structure complexed with a p21-like RE (PDB: 3Q05^14^, Fig. 3b and Supplementary Fig. 6), the Lys120 side chain is disordered, which is a result of its Cα atom within the p53 L1 loop being farther away from the DNA (outside of the major groove), precluding the Lys120 Nζ atom from hydrogen bonding with the purine bases. Arg248 is extended and penetrates into the minor groove undergoing primarily hydrophobic interactions^14,22^. We confirmed that the DNA sequences of the two representative PDB structures, which only differ at 4 bps, recapitulated the characteristics of the two distinct binding modes observed in vitro (Table 1). Thus, p53 indeed adopts structurally distinct binding modes at high-affinity, K120R-bound and low-affinity, K120R-unbound REs.

Although distinct Lys120 and Arg248 conformations have previously been reported in complex with different REs^12,15^, the current findings provide a mechanistic understanding of the roles played by these two key DNA contact residues in providing specificity to genome occupancy by p53. For example, the conversion of Lys120 to arginine exclusively affects the binding affinity to the low-affinity, K120R-unbound REs, since the arginine side chain causes a steric clash with these REs (Fig. 3c). Our analysis also provides a structural basis for the different salt sensitivities observed in vitro (Table 1), as the salt-dependent interaction at low-affinity, K120R-unbound REs can be ascribed to the electrostatic interactions of the four Arg248 residues in tetrameric p53 with the DNA backbone phosphates, while these arginine residues convert to predominantly non-polar, hydrophobic interactions in the salt-insensitive binding mode at high-affinity, K120R-bound REs.

### Minor groove width is sensed by Arg248 and determines the p53 RE-binding mode

Comparison of the DNA sequences in the structurally distinct p53-DNA complexes (Supplementary Table 1) revealed a preponderance of A/T bps in the flanking RRR/YYY regions in the group featuring a disordered Lys120 side chain. Since A/T bps can induce a narrow minor groove and a wider major groove in B-form DNA^24,25^, we examined the shape of the DNA in the representative p53-DNA structures. We found that the width of the major and minor grooves in the regions adjacent to the CWWG core motif—the sites where Arg248 interacts with DNA— are indeed altered in these two structures (Fig. 3d). In A/T-rich, p21-like, K120R-bound RE, the minor groove is narrow and recognized by the extended Arg248 side chain in the primarily hydrophobic mode (Fig. 3b and Supplementary Fig. 6). In G/C-rich, BAX-like, K120R-unbound RE, the minor groove is wider and sensed by Arg248 in a bent conformation, interacting electrostatically with DNA (Fig. 3a and Supplementary Fig. 5). Thus, only two bps adjacent to the CWWG motifs (positions 8 and 18, Fig. 3d) suffice to switch the p53-binding mode in these examples, regardless of the base composition of the CWWG core motif (Supplementary Fig. 7)^11,26^. Our hypothesis that the Arg248 side chain conformation is influenced by minor groove width is confirmed by a double mutation in p53 (R273H/T284R) that widens the minor groove width, resulting in a change of the Arg248 side chain conformation (Supplementary Fig. 8)^27,28^. These findings support the conclusion that minor groove width at bps 3, 8, 13, and 18 in p53 REs is critical in determining different DNA-binding modes because it induces distinct Arg248 conformations and interactions.

### Rewriting the RE code reprograms p53 function

**Fig. 4:**
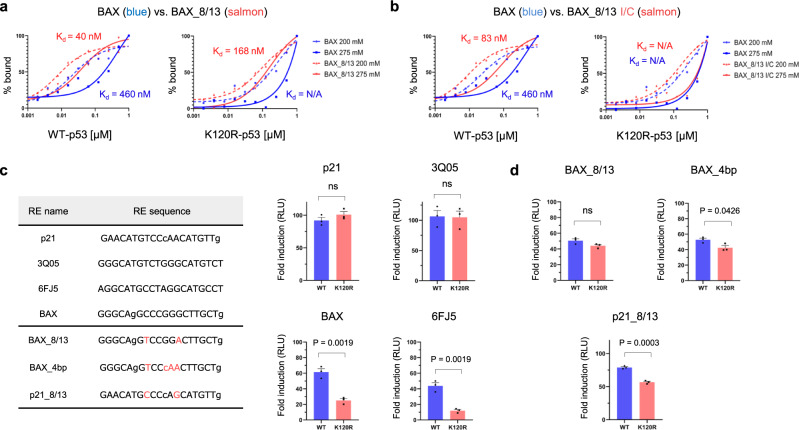
Rewriting the RE code reprograms p53 function in vitro and in cells. **a** G/C to A/T conversions at positions 8 and 13 in BAX RE increase its affinity for WT p53 (from 460 nM to 40 nM, left) and restore K120R-p53 binding (right). **b** G/C to I/C conversions at positions 8 and 13 in BAX RE increase its affinity for WT p53 (from 460 nM to 83 nM, left), but do not have an impact on K120R-p53 binding deficiency (right). Data are shown as graphs depicting the percent of bound protein and plotted as a function of the protein concentration (from 0.001 to 1 μM, in log scale). Two independent measurements were performed at “low-salt” (dashed curves) and “high-salt” (solid curves) buffer conditions. Relevant *K*_d_ values measured at high-salt concentrations are depicted on the graphs. I/C inosine/cytosine. N/A not available. **c** A luciferase reporter plasmid containing p21, 3Q05, 6FJ5, BAX, BAX_8/13, BAX_4bp or p21_8/13 RE (nucleotide sequences are listed in table) were co-transfected with an empty vector, WT or K120R-p53 expression plasmid. Twenty-four hours after transfection cells were harvested and luminescence was measured. K120R-p53 has identical activity as WT p53 protein on A/T-rich p21 and 3Q05 REs, but is defective in trans-activating G/C-rich BAX and 6FJ5 REs. These data confirm K120R-p53 binding behavior observed in vitro. Data are presented as a fold change of measured luminescence in either WT (blue) or K120R (salmon)-p53-expressing cells over luminescence measured in cells transfected with an empty vector (no p53). **d** Substituting G/C to A/T bps in BAX RE (“BAX_8/13”, converted nucleotides are depicted in salmon letters) or changing positions 8–13 from CCCGGG to TCCCAA (“BAX_4bp”) restores K120R-p53 transactivation activity. Conversely, substituting A/T to G/C bps in p21 RE (“p21_8/13”) reintroduces K120R deficiency. Graphs in panels **c** and **d** show cumulative data from three independent experiments (mean ± s.e.m.). Two-tailed unpaired Students *t*-test was used for statistical analysis in panels **c** and **d**. RLU, relative light units. Source data are provided as a Source Data file.

Our model predicts that single or dual bp conversions at positions 3, 8, 13, and/or 18 will impact the minor groove width of an RE, alter interactions of Arg248 and Lys120, and ultimately dictate the binding mode utilized by p53. To rigorously test this model, targeted G/C to A/T, along with A/T to G/C bp conversions were introduced in specific REs in vitro and in vivo. Using the in vitro DNA-binding assay, we measured *K*_d_ values of the modified BAX_8/13 RE that carried G/C to A/T conversions at positions 8 and 13 in a natural BAX RE (Fig. 4a). Consistent with the model, these conversions increased the affinity of WT p53 for this RE (from 460 nM to 40 nM) and restored K120R-p53 binding. An increase in WT and K120R-p53 binding was similarly observed for the NOXA_3/13 RE, which contained G/C to A/T conversions at positions 3 and 13 (Supplementary Fig. 9a). We also measured *K*_d_ values of the p21_8/13 RE, which carried reciprocal A/T to G/C conversions, at positions 8 and 13. As expected, these conversions decreased the affinity of the WT p53 (from 23 nM to 99 nM) and more markedly K120R-p53 (from 43 to 479 nM) for this RE (Supplementary Fig. 9b). Next, we experimentally manipulated the minor groove width of the RE in vitro by introducing G/C to inosine-cytosine (I/C) conversions, which narrows the minor groove without impacting major groove width^29^. These changes are predicted to impact the interaction of Arg248 with DNA by increasing the non-polar nature of the binding while having no impact on K120R sensitivity. Supporting this scenario, WT p53 exhibited an increased affinity for the BAX_8/13 I/C RE (from 460 nM to 83 nM), but K120R-p53 binding remained undetectable (Fig. 4b). Increased WT affinity and retention of K120R binding was also observed after introducing G/C to I/C substitutions at the analogous positions in the artificial 6FJ5 RE (Supplementary Fig. 9c).

To assess whether the RE code could be predictably manipulated in vivo, we examined whether the in vitro binding data correlated with p53-binding behavior in human cells. Initially, this strategy relied on a panel of luciferase reporters driven by specific variants of the p53 RE. Natural and artificial high-affinity and A/T-rich REs (p21 and 3Q05) exhibited full activation by K120R-p53, whereas low-affinity and G/C-rich REs (BAX and 6FJ5) were significantly defective for activation by K120R-p53 (Fig. 4c), in agreement with in vitro results (Table 1). Notably, converting the BAX RE positions 8 and 13 from G/C to A/T or fully converting positions 8 through 13 to resemble the p21 RE (from CCCGGG to TCCCAA, “BAX_4bp”), fully restored K120R-p53 responsiveness. In contrast, A/T to G/C conversions in the p21_8/13 RE decreased activation by K120R-p53, again consistent with the dual-binding mode model proposed here (Fig. 4d).

### Editing the RE code interconverts p53-driven cell fates

As the p53 RE consensus sequence is extremely degenerate, there are few examples in the human genome of active REs, which differ only by a single nucleotide at positions 3, 8, 13, and/or 18. To test our model in the context of native chromatin, this limited number of examples were examined for p53 occupancy. In one of these examples, relying on a comparison of the validated but uncharacterized p53 target genes APBB2 and SSUH2, a dual nucleotide conversion at positions 8 and 13 from A/T bps, in the RE linked to APBB2 gene, to G/C bps, in the RE linked to SSUH2 gene, strongly decreased WT p53 binding and completely diminished binding of K120R-p53 protein, again supporting the model (Supplementary Fig. 10).

**Fig. 5:**
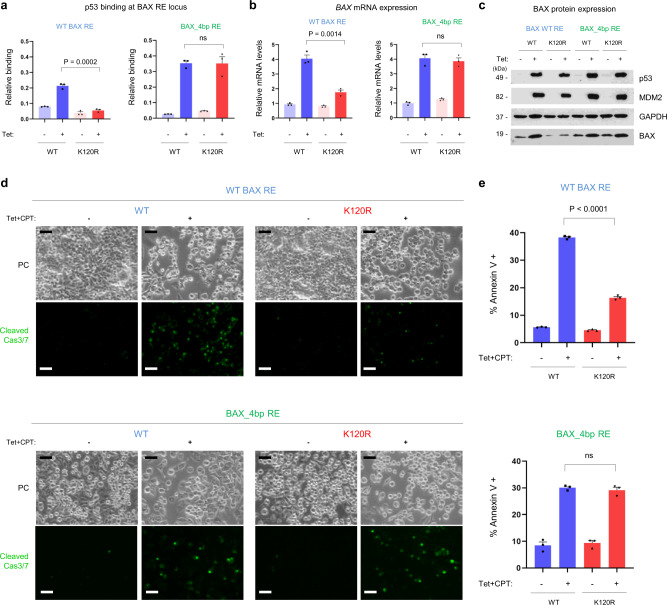
Genomic editing of BAX RE modifies p53 binding and interconverts cell fate outcome. **a** ChIP signals at the genomic BAX RE locus for WT (blue) and K120R (red) p53 in cells with a WT BAX RE (left) or genetically edited BAX_4bp RE (right). Graphs represent precipitated DNA relative to an input (total) DNA and show cumulative data from three independent experiments (mean ± s.e.m.). **b** Quantitative polymerase chain reaction (qPCR) of *BAX* mRNA (represented relative to *GAPDH* mRNA) from the same cells as in panel **a**. The graphs show cumulative data from three independent experiments (mean ± s.e.m.). **c** Representative Western blot from three independent experiments. Blots were probed with antibodies specific for p53, MDM2, GAPDH, and BAX proteins. **d** Same cells as in panels **a**–**c** were treated additionally for 12 h with camptothecin (CPT, 1 μM; to induce DNA damage) and induction of apoptosis was detected by visualizing cleaved Caspase-3/7 by fluorescence microscopy (**d**), or by measuring Annexin V positivity by flow cytometry (**e**). In both assays, a restoration of pro-apoptotic functions of the K120R-p53 variant in cells with edited BAX RE could be observed. Scale bars, 50 μm. PC, phase contrast. Representative images from three independent experiments are shown in panel **d**. Graphs in panel **e** represent an average percentage of Annexin V-positive cells from three independent experiments (mean ± s.e.m.). Two-tailed unpaired Students *t*-test was used for statistical analysis in panels **a**, **b**, and **e**. Source data are provided as a Source Data file.

To formally test the proposed p53 RE code in the context of native chromatin, we utilized CRISPR/Cas9 to edit the sequence of the endogenous BAX RE in human cells. We edited the wild-type BAX RE to increase its A/T content, by converting positions 8, 11, 12, and 13, from CCCGGG to TCCCAA (“BAX_4bp”, identical to the sequence used in the Fig. 4d). Our model predicts that this edited BAX_4bp RE should be recognized by WT p53 through a non-polar, K120R-bound binding mode. Remarkably, when compared to a G/C-rich WT BAX RE, WT p53 exhibited higher occupancy at the edited BAX_4bp RE locus and K120R-p53 binding was completely restored (Fig. 5a and Supplementary Fig. 11a). Furthermore, the restoration of K120R-p53 binding observed by ChIP analysis was of functional relevance as it resulted in rescued BAX mRNA transcription and protein expression, in both WT and K120R-p53-expressing cells (Fig. 5b, c and Supplementary Fig. 11b). Finally, targeted conversions of the BAX RE within native chromatin resulted in a restoration of the pro-apoptotic functions of the cancer-derived K120R-p53 mutant, under conditions where unedited cells undergo cell cycle arrest (Fig. 5d, e)^18^. This data strongly supports the model in vivo and highlights DNA-binding as a crucial step in p53’s ability to selectively activate distinct effector functions. In summary, by introducing this targeted 4-bp change in a BAX RE and converting this binding site from lower affinity, K120R-unbound to higher affinity, K120R-bound, p53-dependent cell fate outcomes were transformed.

## Discussion

Cell fate decisions are frequently regulated by the presence or absence of key transcription factors that control gene expression programs ultimately responsible for the cell phenotype. In some cases, a critical transcription factor can “decide” between multiple cell fate options, depending on the cellular context. For example, in response to different developmental cues, individual members of the SOX and WNT/β-catenin families of transcription factors can drive cell differentiation along several distinct lineage pathways^30,31^. Similarly, the oncogenic transcription factor MYC can trigger either cell cycle progression or cell death, also in a context-dependent manner^32^. Like MYC, the tumor suppressor p53 can trigger multiple cellular outcomes, often depending on the form of stress that initially drove p53 activation^4^. The ability of a single, sequence-specific transcription factor to “choose” to activate only one of several of its gene expression programs raises a number of biochemical questions. Central among these is how recognition of genomic targets linked to different effector functions can be discriminated by a conserved DNA-binding domain. Here, we report a new mechanism of DNA-binding specificity of p53 protein, by showing that a nucleotide code at positions 3, 8, 13, and 18 in the p53 RE creates a DNA shape recognition difference between distinct categories of genomic targets, with these differences being sensed by two key DNA contact residues—Lys120 and Arg248. The code our findings establish is predictive and, when manipulated in vivo, can be utilized to interconvert p53 target gene transcription patterns and cell fate.

As previously mentioned, p53 RE is unusually long and tolerates a substantially higher level of degeneracy than what is typically observed for a sequence-specific DNA-binding protein. Out of twenty nucleotides, G/C bps at positions 4, 7, 14, and 17 (RRRCWWGYYYRRRCWWGYYY) are the only invariant positions in the p53 RE. Guanines at those positions are directly recognized by four Arg280 residues, and this base-specific readout is a well-defined mechanism by which tetrameric p53 recognizes specific genomic targets^33–35^. The number of mismatched bps within a RE, in particular within a core (CWWG) sequence, generally lowers the affinity of individual REs^11^. However, identical numbers of mismatches to the consensus sequence, including mismatches in the crucial G/C bps at positions 4, 7, 14, and 17, are present in both high- and low-affinity-binding sites. This finding suggests that the degree of a mismatch from the consensus RE fails to fully explain the differential binding of p53 to high- and low-affinity REs and that a mechanism beyond base-specific recognition must account for the exquisite ability of p53 to distinguish between different targets.

With base-specific recognition ruled out as the major determinant of RE recognition by p53, other biophysical mechanisms must be considered. Relevant to the findings reported here, minor groove shape recognition is a commonly used mechanism in protein-DNA interactions, utilized by a variety of DNA-binding proteins^24,36^. In particular, recognition of a narrow minor groove by arginine residues has a thermodynamically beneficial impact on protein-DNA interactions, by increasing binding affinity^24,37^. However, differential arginine-minor groove interactions that modulate DNA binding without base-specific recognition, and a DNA-binding protein that uses two binding modes through distinct minor groove interactions, have not been previously described to our knowledge. Our findings identify two distinct molecular mechanisms used by p53 for differential recognition of its genomic targets as a direct consequence of that phenomenon. We find that minor groove width, encoded by the presence of G/C or A/T bps at positions 3, 8, 13, and 18 in the RE, determines the two distinct p53 DNA-binding modes, which are in turn linked to different cell fates.

The RE code proposed here explains initial DNA recognition by p53. However, other factors, such as chromatin structure or central insertions in the RE, may impact or even override this code. For example, discontinuous REs with inserts between two half-sites generally have a lower affinity, independent of their DNA sequence^38^. Furthermore, some genomic sites containing a consensus RE sequence, do not exhibit p53 binding in vivo, perhaps due to a suppressive effect of the aforementioned chromatin landscape^9^. These exceptions provide an important caveat to our model. However, for the vast majority of established p53 targets^10,19^, RE recognition is regulated primarily by DNA sequence, and for these genomic targets, selective DNA-binding, as the first, fundamental step in the activation of downstream p53 targets, can be largely explained via the use of the two distinct DNA-binding modes described in our model.

These two binding modes constitute an additional layer of gene regulation by the p53 protein to create, via Arg248, qualitatively (predominantly hydrophobic vs. predominantly electrostatic) and quantitatively (high-affinity vs. low-affinity) different interactions with distinct classes of REs. This unique role of Arg248 in target gene selectivity builds on its already established and crucial role in p53 biology, most evident from Arg248 being the most frequently mutated residue in human cancer^39^. The observation reported here regarding the ability of Lys120 to sense differences in the RE shape relates to two previous findings of the role this residue plays in DNA recognition and cell fate. First, the Halazonetis group has reported structural findings that the L1 loop, which contains Lys120, can adopt two different conformations when complexed with DNA^14,22^. These conformations are referred to as extended and recessed, reflecting the position of the L1 loop relative to the DNA major groove. Similarly, Ethayathulla et al.^40^ have reported that Lys138, which is the Lys120 analog in the L1 loop of the p53 family member p73, displays different conformations at different REs. The combination of biophysical, genomic, and biological evidence reported here has established a DNA code and p53 recognition mechanisms that unifies these observations. Second, we and others have shown that the Lys120 residue is post-translationally modified by acetylation, whose levels correlate with apoptosis but not cell cycle arrest^16,17^. Ultimately, the acetylation of Lys120 may be a mechanism by which DNA recognition by the p53 is stabilized in one mode or the other and future studies should elucidate its potential role.

In summary, our discovery of two distinct binding modes as a means of conferring p53 RE selectivity through differential recognition of minor groove shape offers an explanation for a major unresolved mystery in p53 biology—the mechanism by which p53 achieves dose-dependent RE recognition and differential target gene activation. Since the existence of four p53 monomers in a complex with four RE quarter-sites allows for a wide range of p53-RE interactions (from strongly polar to strongly non-polar), the two biochemically distinct binding mechanisms identified here allow p53 to impose a subtle “fine-tuning” of its DNA recognition. Additionally, our model describes DNA recognition by p53 via two distinct binding modes as initial and foundational step in p53-dependent activation of downstream targets. Other factors, such as the specific stress signaling pathway responsible for p53 activation, post-translational modification of residues beyond Lys120 in the p53 DNA binding domain or elsewhere in the protein, differential co-factor binding, or pulsatile expression dynamics, are likely to play a role in modulating selective target gene activation by p53 and enacting an ultimate cell fate outcome^4^. Having established a paradigm for a single transcription factor using different DNA-binding modes determined by different arginine-minor groove interactions to selectively effect distinct downstream pathways, future studies may identify other DNA-binding proteins involved in cell fate decisions, that use similar mechanisms to gain target gene selectivity.

## Methods

### Cell culture, creation of stable cell lines, and transient transfections

The p53-null human lung cancer cell line H1299 (ATCC) was cultured in Dulbecco’s Modified Eagle Medium (DMEM, Corning) supplemented with 10% fetal bovine serum (FBS, GeminiBio) at 37 °C and 5% CO2. Tetracycline (Tet)-inducible p53-expressing stable cell lines (H1299To-p53) were generated via lentiviral infection using a ViraPower HiPerform Lentiviral Expression System (Invitrogen)^18^. p53 expression vectors were generated in pLenti6.3/V5-DEST cloning vectors (Invitrogen) and p53 protein expression was induced by treatment of tetracycline repressor (TetR)-expressing H1299 cells with 1 μg/ml of Tetracycline (Millipore Sigma). Expression vectors encoding for Lys120 to Arg mutants of p53 (K120R) were generated using the QuikChange XL Site-Directed Mutagenesis kit (Agilent). For transient protein expression, parental H1299 cells were transfected with p53 expression vectors, generated in a pcDNA3.1 cloning vector (Addgene) and harvested 24 h after transfection. All transfections were performed using Lipofectamine 2000 (Invitrogen), according to manufacturer’s instructions. Cell lines were regularly tested and verified to be mycoplasma negative using LookOut Mycoplasma PCR Detection Kit (Sigma-Aldrich).

### Western blotting

After harvesting, cells were lysed in an E1A-style cell-lysis buffer [20 mM NaH2PO4, 150 mM NaCl, 50 mM NaF, 0.5% (w/v) IGEPAL, 2.5 mM EDTA, 125 mM sodium pyruvate, and 10% (w/v) glycerol]^17^, supplemented with a proteinase inhibitor cocktail (1:1000, Millipore Sigma). After 30 min on ice, lysates were centrifuged at 20,000 × *g* for 10 min at 4 °C to remove cell debris. Protein concentrations in lysates were determined using the Pierce BCA Protein Assay Kit (ThermoFisher Scientific) according to manufacturer’s instructions. Cell lysates were then boiled with sodium dodecyl sulfate (SDS) loading buffer (250 mM Tris–HCl (pH 6.8), 8% (w/v) sodium dodecyl sulfate, 0.2% (w/v) bromophenol blue, 40% (v/v) glycerol, 20% (v/v) β-mercaptoethanol) for 10 min, separated by SDS–polyacrylamide gel electrophoresis (SDS-PAGE) and electroblotted onto nitrocellulose membranes. After blotting, membranes were blocked in 3–5% dry milk (AppliChem) in TBS-T [20 mM Tris, 150 mM NaCl, 0.1% Tween (Millipore Sigma)] and incubated with primary antibodies (Supplementary Table 2). After an overnight incubation at 4 °C, membranes were washed three times in TBS-T before 1–2 h incubation with a mouse and/or rabbit secondary antibodies (Supplementary Table 2). After the final wash, proteins were detected using a chemiluminescence reagent (Thermo Scientific).

### RNA analysis and chromatin-immunoprecipitation

Total RNA was isolated using TRIzol (Thermo Fisher) according to manufacturer’s instructions. cDNA was generated from 200 ng of RNA using the High Capacity cDNA Reverse Transcription Kit (Thermo Fisher). For reverse transcription polymerase chain reactions (RT-PCRs), 2 μl of cDNA was used per reaction. Quantification of DNA was performed using the Step One Plus Real Time-PCR system (v2.3) with Fast SYBR Green Master Mix (both Applied Biosystems). Gene values were normalized to *GAPDH* gene expression. Primer sequences for each gene are listed in Supplementary Table 3.

Chromatin-immunoprecipitation, including cross-linking, cell lysis, sonication, immunoprecipitation, purification, and PCR were performed as previously described^41^ using anti-p53 antibody (rabbit monoclonal, sc-6243 X, Santa Cruz Biotechnology). ChIP products were quantified using the Step One Plus RT-PCR system (v2.3) and Fast SYBR Green Master Mix (both Applied Biosystems). Each immunoprecipitation was normalized to the amount of DNA detected in the input sample by RT-PCR. The list of primers used for PCR reactions can be found in the Supplementary Table 4.

### Chromatin-immunoprecipitation sequencing (ChIP-seq) data generation and analysis

The chromatin-immunoprecipitation sequencing (ChIP-seq) experiments were performed in H1299To-p53 cells treated with Tet for 24 h, as previously described^41^, using the anti-p53 antibody (rabbit monoclonal, sc-6243 X, Santa Cruz Biotechnology). Sequencing libraries were prepared using Accel-NGS 2S Plus Library Kit (Swift Biosciences) with unique single indexes. Library insert size and molarity was quantified using the BioAnalyzer (Agilent) and KAPA Library Quantification methods. Multiplexed libraries were sequenced on the NextSeq 500 System (Illumina) with V1 chemistry to generate 75-bp reads. Uniquely aligned reads were aligned to the GRCh37/hg19 reference genome using BWA-MEM (v0.7.12)^42^ and enriched regions were determined using MACS (v2)^43^. Bed files for visualization on the UCSC Genome Browser were generated using HOMER (v4.10)^44^ with a visualization fragment length equal to the median estimated fragment length. 20-bp-long p53 response element sequences were obtained from a previously published list of DNA sequences of all natural p53-binding sites by aligning chromosomal locations of our ChIP-seq-generated peaks to 20 bp-long ChIP-Exo-generated p53-binding sites by Chang et al.^10^. DNA logos were created using WebLogo (v3)^45^.

### Protein purification

Untagged, thermostable, N-terminally truncated human p53 (residues 94-393)^14,22^ and its Lys120 to Arg (K120R) mutant were expressed in BL21-CodonPlus (DE3)-RIL E. coli (Stratagene). Typically, 6 L of bacteria cell cultures were grown at 37 °C until they reached an OD600 = 0.5–0.8 when they were shifted to 18 °C. Protein expression was induced by addition of 0.2 mM isopropyl-β-d-thiogalactoside (IPTG). Cells were harvested by centrifugation (20 min, at 7500 × *g* and 4 °C), resuspended in cell lysis buffer [20 mM bis-tris propane (BTP, pH 6.8), 200 mM NaCl, 2 mM DTT, and 0.5 mM Tris (2-carboxyethyl) phosphine hydrochloride (TCEP)] and homogenized by an Emulsiflex C5 cell disruptor (Avestin). Cell lysates were centrifuged at 35,000 × *g* for 30 min, at 4 °C and cleared cell extracts were loaded onto a HiTrap SP cation exchange column (GE Healthcare) pre-equilibrated with a lysis buffer. The proteins were eluted using a linear gradient of NaCl from 200 mM to 1 M concentration. Eluted proteins were then loaded onto a HiTrap Q anion exchange column (GE Healthcare) to remove residual nucleic acids, and the protein-containing flow through was collected. Finally, the pooled protein was concentrated and loaded onto a HiLoad Superdex 200 16/60 size exclusion column (GE Healthcare) and eluted in a buffer containing 150 mM NaCl, 20 mM BTP (pH 6.8), and 0.5 mM TCEP. Final protein concentrations were estimated by measuring absorbance at 280 nm.

### Steady-state fluorescence-based DNA binding assay

Fluorescence polarization measurements were carried out at 25 °C on a VICTOR3V plate reader (Perkin Elmer), as previously described^20^. The 6-carboxy-fluorescein (FAM)-labeled dsDNA probes (2.5 nM) were incubated for 10 min with increasing amounts of protein up to 1 μM in a buffer containing 200 or 275 mM NaCl, 20 mM BTP (pH 6.8), 5% (v/v) glycerol, 0.1 mg/ml BSA, and 0.5 mM TCEP. The 36-bp oligonucleotides used in DNA-binding assays are listed in the Supplementary Table 5. No change in fluorescence intensity was observed with the addition of protein. Binding curves were fit individually using GraphPad Prism (v8.4.2, GraphPad Software, Inc.). Binding constants (*K*_d_ values) were obtained from curve fitting using the following formula for millipolarization (mP): [mP] = [maximum mP] × [C]/(*K*_d_ + [C]) + [baseline mP], where [C] is the protein concentration. Normalized [mP] values were calculated from {([mP] − [baseline mP])/([maximum mP] − [baseline mP])} × 100, and plotted as “% bound”. Averaged *K*_d_ values are reported (*n* = 2).

### Crystallographic analysis

All coordinates and structural factors were retrieved from Protein Data Bank (PDB) (https://www.rcsb.org/). Simulated annealing omit maps were calculated by PHENIX (v1.6)^46^. Major and minor groove widths were measured by 3xDNA (v2)^47,48^. Molecular graphics were generated using PyMol (v2.3, DeLano Scientific, LLC).

### Luciferase reporter assay

pGL3 firefly luciferase reporter plasmids encoding for natural (p21 and BAX) REs have been described previously^49^. Reporter plasmids with mutated p53 REs were generated using the QuikChange II Site-Directed Mutagenesis kit (Agilent), according to manufacturer’s instructions. A list of all RE sequences used in Luciferase reporter assays is included in Fig. 4c. Briefly, 500 ng of Firefly luciferase reporter plasmids were co-transfected with 250 ng of p53 expression vectors and 100 ng of pRL-TK Renilla luciferase control reporter vectors (Promega), using Lipofectamine 2000 (Invitrogene), according to manufacturer’s instructions. Cells were harvested 24 h post transfection and relative luciferase activity, in terms of relative luminometer units (RLU), was measured using the Dual-Luciferase Reporter Assay System (Promega), using Infinite 200 Pro (v3.37, Tecan). All data is presented as fold induction over the empty vector control with standard deviations from three independent experiments.

### CRISPR/Cas9-mediated editing of the BAX RE in human cells

The wild-type response element within the intronic region of BAX gene (RefSeq NG_012191.1) was examined for available *Streptococcus pyogenes* (Sp) Cas9 guide RNA (gRNA) sites and a gRNA with the 5’- GTGGTGCGGGCGACAAGCCC sequence was chosen. To drive the homology directed repair (HDR), a 200-mer single-stranded oligonucleotide (Supplementary Table 6) complementary to the plus-strand, centered over the intended mutation site, and with targeted four nucleotide mismatches was synthesized. In all, 50 pmol of purified SpCas9 and 250 pmol of gRNA were incubated at room temperature for 20 min in 1x Tris-EDTA (TE) buffer, prior to transfection. Human H1299 cells were cultured in DMEM (Corning) with 10% FBS (GeminiBio). Transfections were performed using a 4D-Nucleofector (Lonza). Cells, the prepared RNP and 6 μM of single-stranded HDR template were transfected in a 100 μl reaction utilizing the Lonza SF solution and DS-138 program. After transfection, the cells were returned to culture for 72 h to recover. The cells were then single-cell sorted into 96-well plates using manual limited dilutions. Expanded clones were sampled and genomic DNA was purified for PCR amplification. Samples were then sequenced across the BAX RE region (SeqStudio, Applied Biosystems) in both directions using the PCR primers (primer sequences are listed in Supplementary Table 5). Clones with the desired 4-bp mutation (termed “BAX_4 bp”) were identified by alignment to the BAX gene sequence.

### Creation of Tet-inducible, p53-expressing, CRISPR/Cas9-edited stable cell lines

After identification and expansion of the desired 4-bp mutation clones, Tet-inducible p53-expressing, “BAX-4bp” stable H1299 cell lines were generated as previously described, via lentiviral infection using a ViraPower HiPerform Lentiviral Expression System (Invitrogen)^18^. WT and K120R-p53 protein expression was induced by treatment of TetR-expressing “BAX_4bp” cells with 1 μg/ml of Tetracycline (Millipore Sigma).

### Annexin V staining

Following treatment, cells were collected by trypsinization (Corning), washed three times with phosphate buffer saline (PBS, Corning), and stained for Annexin V, using an Annexin V-FITC apoptosis detection kit (BD PharMingen), for 45 min, at room temperature, as previously described^50^. The percentage of Annexin V-positive cells was determined by flow cytometry on a CytoFlex, using the CytExpert (v1.2, both Beckman Coulter). Reported are average values with standard errors, in a population of 10,000 cells, from three independent experiments. The original source images for a representative data and Annexin V gating strategies can be found in the Supplementary Fig. 12.

### Cleaved Cas3/7 fluorescent dye staining and visualization

To visualize the induction of apoptosis, cells were stained with the IncuCyte Caspase-3/7 Green Reagent (Sartorius), according to manufacturer’s instructions. Following treatment, cell culture media were incubated with green fluorescent dye for 24 h, and live-cell images of cells undergoing caspase-3/7-mediated apoptosis were acquired by fluorescence contrast and phase contrast microscopy on an Axio Vert.A1 inverted microscope, equipped with the Axiocam ICm 1 camera, and microscopic images were generated using ZEN (v2.3, all Zeiss). Reported images are representative of one experiment with at least three independent biological replicates.

### Statistical analysis

All statistical analyses were performed with GraphPad Prism software (v.8.4.2, GraphPad Software, Inc.) and Microsoft Excel 2010. Statistical differences between two groups were assessed using a two-tailed Student’s *t*-test. *P* < 0.05 was the cutoff used for statistical significance and exact *P*-value was reported where available.

### Reporting summary

Further information on research design is available in the Nature Research Reporting Summary available in Supplementary Information linked to this article.

## Supplementary information

Supplementary Information

Reporting Summary

## Data Availability

The data supporting the study are available within the paper and its Supplementary Information files. ChIP-sequencing data generated during the study have been deposited in the Gene Expression Omnibus (GEO) repository and are available under GEO accession number GSE159945. UCSC Genome Browser session for the visualization of generated ChIP-sequencing data is available from the corresponding author on request. Structural figures of p53 in a complex with different DNA were generated from publicly available datasets from the Protein Data Bank (3Q05 [DOI: 10.2210/pdb3q05/pdb]; 6FJ5 [DOI: 10.2210/pdb6FJ5/pdb]; 4HJE [DOI: 10.2210/pdb4HJE/pdb]; 4IBW [DOI: 10.2210/pdb4IBW/pdb]; 3IGL [DOI: 10.2210/pdb3IGL/pdb]. Source Data are provided with this paper.

## Source data

Source Data

## References

[1] Levine AJ, Oren M (2009). The first 30 years of p53: growing ever more complex. Nat. Rev. Cancer.

[2] Kastenhuber ER, Lowe SW (2017). Putting p53 in context. Cell.

[3] El-Deiry WS, Kern SE, Pietenpol JA, Kinzler KW, Vogelstein B (1992). Definition of a consensus binding site for p53. Nat. Genet.

[4] Hafner A, Bulyk ML, Jambhekar A, Lahav G (2019). The multiple mechanisms that regulate p53 activity and cell fate. Nat. Rev. Mol. Cell Biol..

[5] Szak ST, Mays D, Pietenpol JA (2001). Kinetics of p53 binding to promoter sites in vivo. Mol. Cell. Biol..

[6] Inga A, Storici F, Darden TA, Resnick MA (2002). Differential transactivation by the p53 transcription factor is highly dependent on p53 level and promoter target sequence. Mol. Cell Biol..

[7] Jackson JG, Pereira-Smith OM (2006). p53 is preferentially recruited to the promoters of growth arrest genes *p21* and *GADD45* during replicative senescence of normal human fibroblasts. Cancer Res..

[8] Veprintsev DB, Fersht AR (2008). Algorithm for prediction of tumour suppressor p53 affinity for binding sites in DNA. Nucleic Acids Res..

[9] Su D (2015). Interactions of chromatin context, binding site sequence content, and sequence evolution in stress-induced p53 occupancy and transactivation. PLoS Genet.

[10] Chang GS (2014). A comprehensive and high-resolution genome-wide response of p53 to stress. Cell Rep..

[11] Wang B, Xiao Z, Ren EC (2009). Redefining the p53 response element. Proc. Natl Acad. Sci. USA.

[12] Kitayner M (2006). Structural basis of DNA recognition by p53 tetramers. Mol. Cell.

[13] Pan Y, Nussinov R (2010). Lysine120 interactions with p53 response elements can allosterically direct p53 organization. PLoS Comput. Biol..

[14] Petty TJ (2011). An induced fit mechanism regulates p53 DNA binding kinetics to confer sequence specificity: p53 conformational switch. EMBO J..

[15] Lukman S, Lane DP, Verma CS (2013). Mapping the structural and dynamical features of multiple p53 DNA binding domains: insights into loop 1 intrinsic dynamics. PLoS ONE.

[16] Tang Y, Luo J, Zhang W, Gu W (2006). Tip60-dependent acetylation of p53 modulates the decision between cell-cycle arrest and apoptosis. Mol. Cell.

[17] Sykes SM (2006). Acetylation of the p53 DNA-binding domain regulates apoptosis induction. Mol. Cell.

[18] Monteith JA (2016). A rare DNA contact mutation in cancer confers p53 gain-of-function and tumor cell survival via TNFAIP8 induction. Mol. Oncol..

[19] Fischer M (2017). Census and evaluation of p53 target genes. Oncogene.

[20] Hashimoto H (2014). Wilms tumor protein recognizes 5-carboxylcytosine within a specific DNA sequence. Genes Dev..

[21] Arbely E (2011). Acetylation of lysine 120 of p53 endows DNA-binding specificity at effective physiological salt concentration. Proc. Natl Acad. Sci..

[22] Emamzadah S, Tropia L, Vincenti I, Falquet B, Halazonetis TD (2014). Reversal of the DNA-binding-induced Loop L1 conformational switch in an engineered human p53 protein. J. Mol. Biol..

[23] Golovenko D (2018). New insights into the role of DNA shape on its recognition by p53 proteins. Structure.

[24] Rohs R (2009). The role of DNA shape in protein–DNA recognition. Nature.

[25] Patel A (2018). DNA conformation induces adaptable binding by tandem zinc finger proteins. Cell.

[26] Chen Y (2013). Structure of p53 binding to the BAX response element reveals DNA unwinding and compression to accommodate base-pair insertion. Nucleic Acids Res..

[27] Eldar A, Rozenberg H, Diskin-Posner Y, Rohs R, Shakked Z (2013). Structural studies of p53 inactivation by DNA-contact mutations and its rescue by suppressor mutations via alternative protein-DNA interactions. Nucleic Acids Res..

[28] Kitayner M (2010). Diversity in DNA recognition by p53 revealed by crystal structures with Hoogsteen base pairs. Nat. Struct. Mol. Biol..

[29] Lang D, Stamminger T (1994). Minor groove contacts are essential for an interaction of the human cytomegalovirus IE2 protein with its DNA target. Nucleic Acids Res..

[30] Metz EP, Rizzino A (2019). Sox2 dosage: A critical determinant in the functions of Sox2 in both normal and tumor cells. J. Cell Physiol..

[31] Ramalingam H (2019). Disparate levels of beta-catenin activity determine nephron progenitor cell fate. Dev. Biol..

[32] McMahon SB (2014). MYC and the control of apoptosis. Cold Spring Harb. Perspect. Med..

[33] Garvie CW, Wolberger C (2001). Recognition of specific DNA sequences. Mol. Cell.

[34] Pabo CO, Sauer RT (1992). Transcription factors: structural families and principles of DNA recognition. Annu. Rev. Biochem..

[35] Cho Y, Gorina S, Jeffrey PD, Pavletich NP (1994). Crystal structure of a p53 tumor suppressor-DNA complex. Science.

[36] Slattery M (2014). Absence of a simple code: how transcription factors read the genome. Trends Biochem. Sci..

[37] Chiu T-P, Rao S, Mann RS, Honig B, Rohs R (2017). Genome-wide prediction of minor-groove electrostatic potential enables biophysical modeling of protein–DNA binding. Nucleic Acids Res..

[38] Vyas P (2017). Diverse p53/DNA binding modes expand the repertoire of p53 response elements. Proc. Natl Acad. Sci. USA.

[39] Bouaoun L (2016). *TP53* variations in human cancers: new lessons from the IARC TP53 database and genomics data: human mutation. Hum. Mutat..

[40] Ethayathulla AS (2012). Structure of p73 DNA-binding domain tetramer modulates p73 transactivation. Proc. Natl Acad. Sci USA.

[41] Schmidt D (2009). ChIP-seq: Using high-throughput sequencing to discover protein–DNA interactions. Methods.

[42] Li, H. Aligning sequence reads, clone sequences and assembly contigs with BWA-MEM. Preprint at arXiv:1303.3997 [q-bio] (2013).

[43] Zhang Y (2008). Model-based analysis of ChIP-Seq (MACS). Genome Biol..

[44] Heinz S (2010). Simple combinations of lineage-determining transcription factors prime cis-regulatory elements required for macrophage and B cell identities. Mol. Cell.

[45] Crooks GE (2004). WebLogo: a sequence logo generator. Genome Res..

[46] Afonine PV (2012). Towards automated crystallographic structure refinement with *phenix.refine*. Acta Crystallogr. D. Biol. Crystallogr..

[47] Lu X-J (2003). 3DNA: a software package for the analysis, rebuilding and visualization of three-dimensional nucleic acid structures. Nucleic Acids Res..

[48] Colasanti AV, Lu X-J, Olson WK (2013). Analyzing and building nucleic acid structures with 3DNA. J. Vis. Exp..

[49] Resnick-Silverman L, St. Clair S, Maurer M, Zhao K, Manfredi JJ (1998). Identification of a novel class of genomic DNA-binding sites suggests a mechanism for selectivity in target gene activation by the tumor suppressor protein p53. Genes Dev..

[50] Patel JH, McMahon SB (2006). Targeting of Miz-1 is essential for Myc-mediated apoptosis. J. Biol. Chem..

