# Supplementary material for: Distinct mechanisms control genome recognition by p53 at its target genes linked to different cell fates
Source: Nat Commun. 2021 Jan 20;12:484. doi: 10.1038/s41467-020-20783-z (PMC7817693; doi:10.1038/s41467-020-20783-z)
Supplement: Supplementary file 1 — Supplementary Information [file 41467_2020_20783_MOESM1_ESM.pdf]

Supplementary Information

Distinct mechanisms control the interaction of p53 with genomic targets linked to different cell fates

Farkas et al.

The Supplementary Information contains:

- Supplementary Figs. 1-12
- Supplementary Tables 1-6
- Supplementary References

Supplementary Figures:

- Supplementary Figs. 1-11, which provide additional experimental data related to Figures 1-5 in the main text.
- Supplementary Fig. 12: The original source images for a representative flow cytometry data, related to Fig. 5e.

Supplementary Tables:

- Supplementary Table 1, related to Figure 3 in the main text.
- Supplementary Table 2: Antibodies used in this paper
- Supplementary Table 3: cDNA primers for qRT-PCR used in this paper
- Supplementary Table 4: ChIP primers used in this paper
- Supplementary Table 5: Sequences of fluorescently labelled 36-bp DNA oligos used in FP assays
- Supplementary Table 6: DNA oligos used for CRISPR/Cas9-mediated genome editing

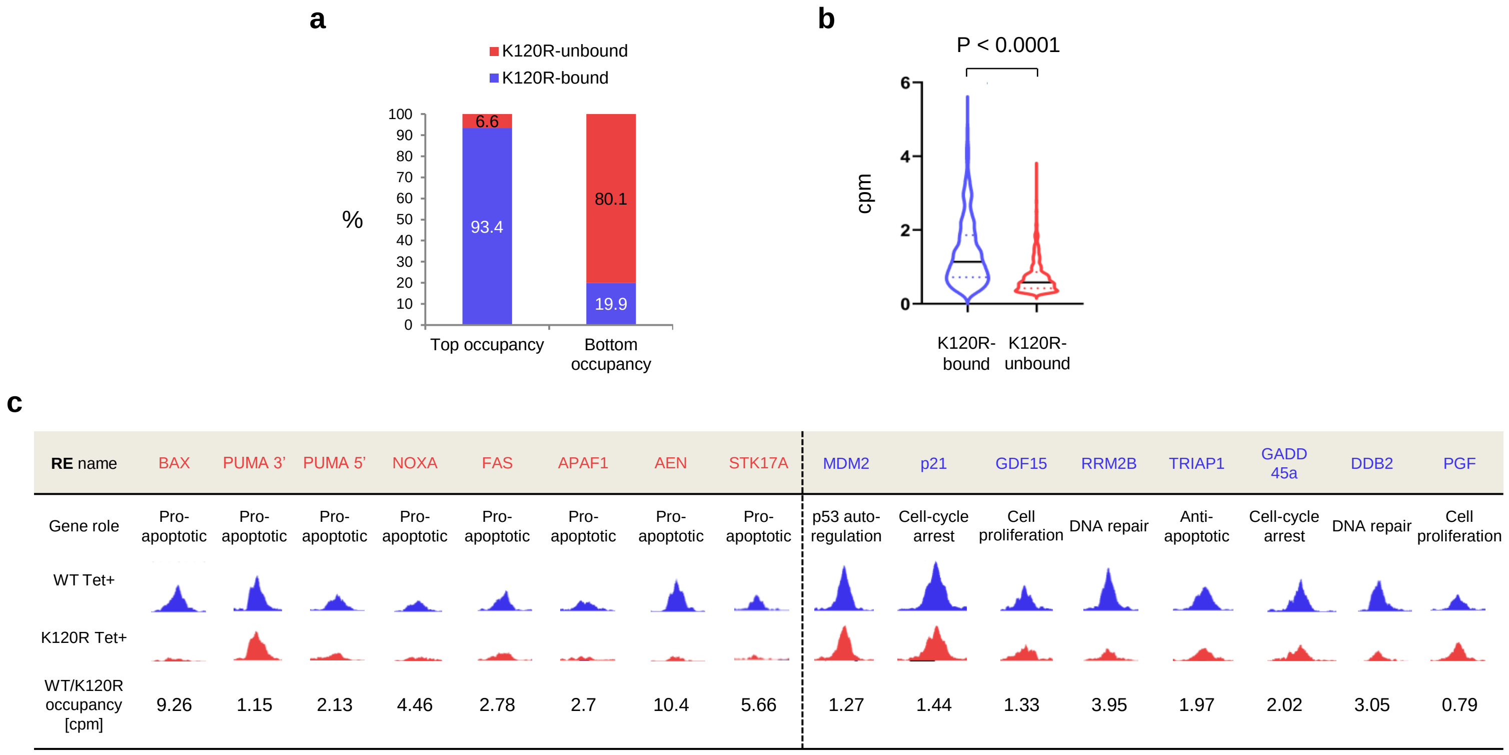

Supplementary Fig. 1: K120R mutation diminishes p53 binding at lower occupancy binding sites, including REs at the vast majority of pro-apoptotic targets, related to Fig. 1. a, Percentage of K120R-bound and K120R-unbound binding sites amongst peaks with the top or bottom decile WT p53 occupancy (for both groups, n = 136 uniquely identified peaks), showing that only ~6% of the peaks in the "Top occupancy" group were K120R-unbound, in contrast to ~80% of the peaks from the "Bottom occupancy" group. **b,** WT p53 occupancy amongst K120R-bound (n = 622) and K120R-unbound (n = 736) uniquely identified peaks, highlighting the generally lower occupancy at REs sensitive to K120R mutation. Full lines depict mean values, dashed lines depict lower and upper quartiles. Two-tailed unpaired Students t-test was used for statistical analysis. **c,** UCSC Genome Browser track screenshots of the WT (blue) and K120R (salmon) p53 binding after Tet-treatment on eight REs at pro-apoptotic genes (left) and eight REs at genes driving p53-dependent pro-survival response (right)[1], showing that K120R mutation diminishes p53 binding at all but one (PUMA 3') pro-apoptotic REs. In many cellular contexts, the target gene *PUMA* participates in p53-mediated apoptosis, with BAX being the downstream effector of PUMA[2]. Since p53 has both K120R-bound (PUMA 3') and K120R-unbound (PUMA 5') REs at the *PUMA* gene, and the previous finding showed that HCT116 cells undergo p53-mediated apoptosis even when the *PUMA* gene is deleted[3], our subsequent analysis focused on the BAX RE as a representative pro-apoptotic, K120R-unbound RE. The last row depicts the WT over K120R peak occupancy (in cpm), highlighting a bigger impact of the K120R mutation on p53's binding at analyzed pro-apoptotic REs (an average ratio of ~4.8 for pro-apoptotic REs vs. ~1.9 for pro-survival REs). Cpm, counts per million (reads). Source data are provided as a Source Data file.

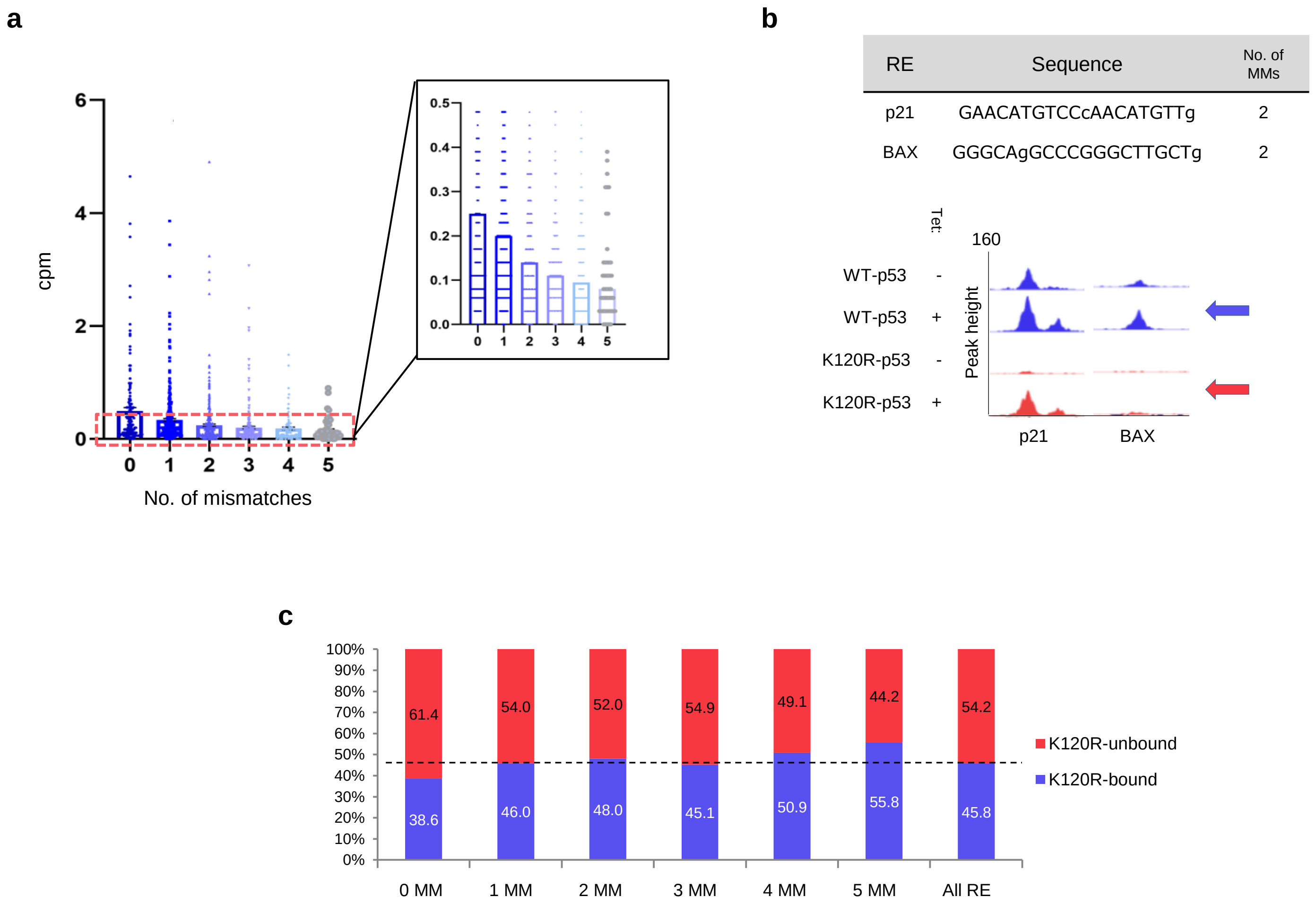

Supplementary Fig. 2: The generic number of mismatches does not (solely) regulate *in vivo* occupancy by p53 at certain REs, or their K120R sensitivity, related to Fig. 1. a, Peak occupancy values (in cpm) of the WT p53 at REs with 0 (*n* = 153 uniquely identified peaks), 1 (*n* = 346 peaks), 2 (*n* = 400 peaks), 3 (*n* = 246 peaks), 4 (*n* = 114 peaks) or 5 mismatches (*n* = 52 peaks) from the consensus sequence. Data is presented as a scatter plot, depicting all individual peak occupancy values. Mean value (± s.e.m.) of each group is presented with the full line. Dashed box highlights the existence of numerous REs with a different number of mismatches, but very similar occupancy. Zoomed-in image of those peaks is shown on the right from the original graph. **b,** Sequences of REs at *p21* and *BAX* genes, as an example of binding sites with the same number of mismatches (in lower case letters), but very different WT (blue arrow) and K120R (red arrow) p53 binding (as shown in Fig. 1c). **c**, Percentage of K120R-bound vs. K120R-unbound REs within groups of REs with 0 (*n* = 153 uniquely identified peaks), 1 (*n* = 346 peaks), 2 (*n* = 400 peaks), 3 (*n* = 246 peaks), 4 (*n* = 114 peaks) or 5 mismatches (*n* = 52 peaks), showing that there is no increase in K120R sensitivity with an increase in number of mismatches. The dashed line presents the percentage amongst all WT p53 binding sites (*n* = 1,358 uniquely identified peaks in our ChIP-seq experiment). MM, mismatch. Cpm, counts per million (reads). Source data are provided as a Source Data file.

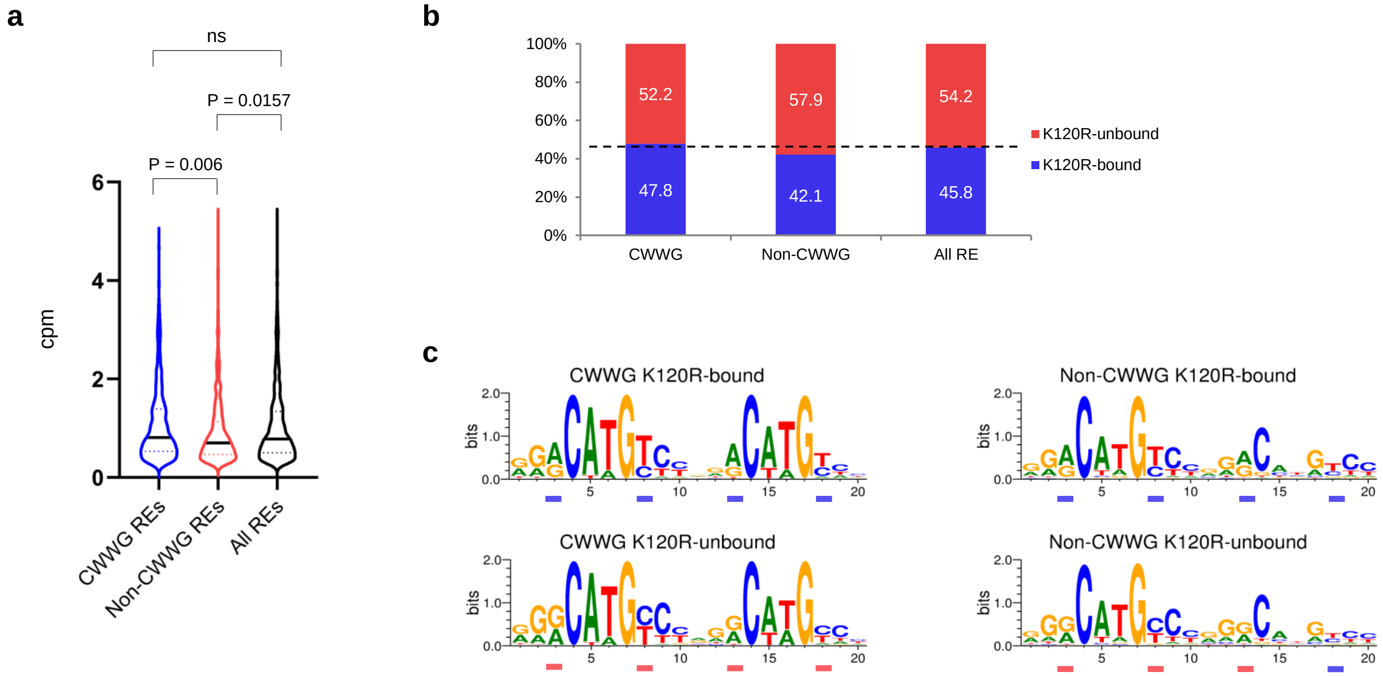

Supplementary Fig. 3: The mismatches within core (CWWG) sequence slightly decrease, but do not (solely) regulate *in vivo* occupancy by p53 at individual REs, or their K120R sensitivity, related to Fig. 2. a, Peak occupancy values (in cpm) of the WT p53 at REs with no mismatches in CWWG sequences ("CWWG REs", *n* = 895 uniquely identified peaks) and REs with at least one mismatch in a CWWG core sequence ("Non-CWWG REs", *n* = 463 uniquely identified peaks). Full lines depict mean values, dashed lines depict lower and upper quartiles. Two-tailed unpaired Students *t*-test was used for statistical analysis of differences in peak heights. **b,** Percentage of K120R-bound vs. K120R-unbound REs within groups of REs with "perfect" CWWG sequences ("CWWG") and REs with CWWG sequences with at least one mismatch ("Non-CWWG")**.** The dashed line presents the percentage amongst all WT p53 binding sites (*n* = 1,358 peaks), highlighting no significant increase in K120R-sensitivty amongst "Non-CWWG" REs. **c**, Positional analysis confirms an increase of G/C bps (salmon-colored ticks) at positions 3, 8, 13, and 18 amongst K120R-unbound REs, and A/T bps (blue ticks) at those positions in K120R-bound sites at both CWWG-containing sites (left panels), and non-CWWG-containing sites (right panels). W = A or T. Cpm, counts per million (reads). Source data are provided as a Source Data file.

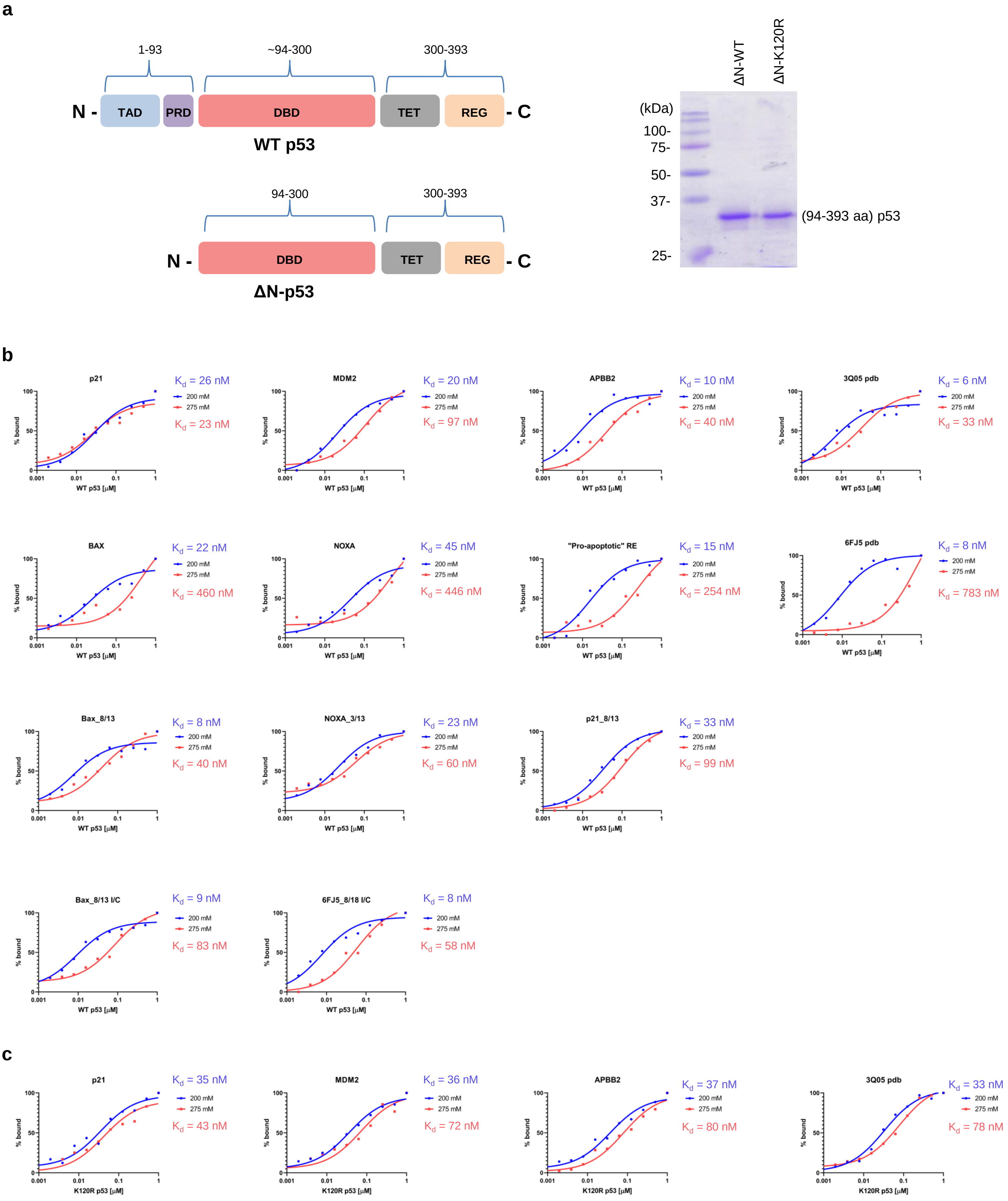

a
1-93
~94-300
300-393
N -
TAD
PRD
DBD
TET
REG
- C
WT p53
94-300
300-393
N -
DBD
TET
REG
- C
ΔN-p53
ΔN-WT
ΔN-K120R
(kDa)
100-
75-
50-
37-
25-
(94-393 aa) p53
b
p21
K_d = 26 nM
K_d = 23 nM
MDM2
K_d = 20 nM
K_d = 97 nM
APBB2
K_d = 10 nM
K_d = 40 nM
3Q05 pdb
K_d = 6 nM
K_d = 33 nM
BAX
K_d = 22 nM
K_d = 460 nM
NOXA
K_d = 45 nM
K_d = 446 nM
"Pro-apoptotic" RE
K_d = 15 nM
K_d = 254 nM
6FJ5 pdb
K_d = 8 nM
K_d = 783 nM
Bax_8/13
K_d = 8 nM
K_d = 40 nM
NOXA_3/13
K_d = 23 nM
K_d = 60 nM
p21_8/13
K_d = 33 nM
K_d = 99 nM
Bax_8/13 I/C
K_d = 9 nM
K_d = 83 nM
6FJ5_8/18 I/C
K_d = 8 nM
K_d = 58 nM
200 mM
275 mM
% bound
WT p53 [μM]
c
p21
K_d = 35 nM
K_d = 43 nM
MDM2
K_d = 36 nM
K_d = 72 nM
APBB2
K_d = 37 nM
K_d = 80 nM
3Q05 pdb
K_d = 33 nM
K_d = 78 nM
200 mM
275 mM
% bound
K120R p53 [μM]

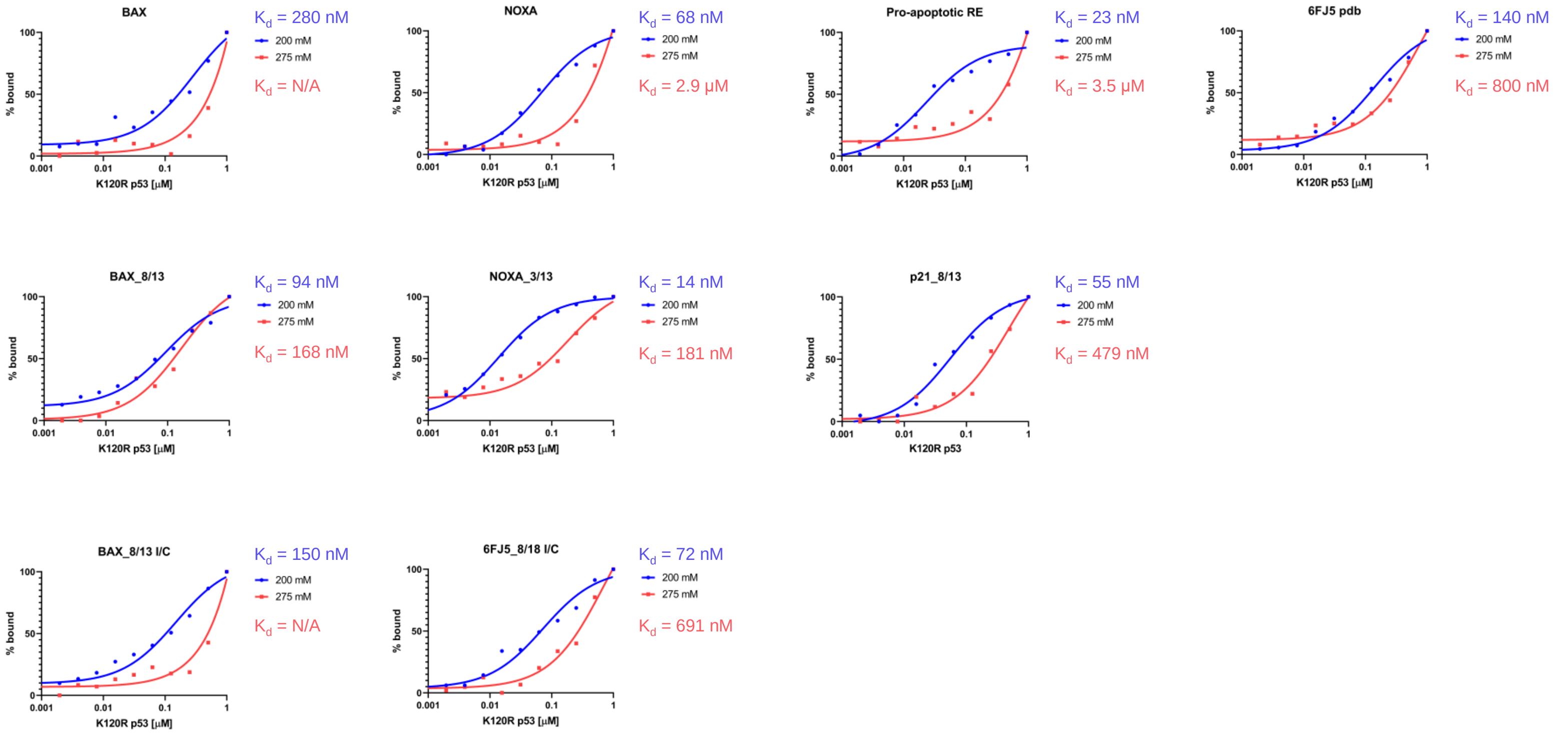

Supplementary Fig. 4: Additional data from *in vitro* fluorescence polarization DNA-binding assays, related to Table 1. a, Left: Schematic of the ΔN-p53 protein construct[4,5] used for DNA-binding experiments, lacking the acidic N-terminal transactivation and proline-rich regions of a full-length protein. Right: ~35 kDa WT and K120R-p53 protein constructs were detected by Coomassie Brilliant Blue staining. Representative image of 3 independent experiments is shown. **b, c,** Graphs depicting the binding of fluorescently labeled oligonucleotides (full sequences can be found in Supplementary Table 4) to WT (**b**) and K120R-p53 (**c**) protein constructs. Percent of protein-bound is plotted as a function of a protein concentration (from 0.001 to 1 µM, in log scale). Two independent measurements were performed under "high-salt" (275mM NaCl, pink curves) and "low-salt" (200mM NaCl, blue curves) buffer conditions. Calculated K_d values (n = 2 independent measurements) are reported on graphs and in Table 1. Source data are provided as a Source Data file.

Supplementary Table 1: List of p53-DNA crystal structures available in Protein Data Bank (PDB), classified according to the omit electron density of Lys120 side chain, related to Fig. 3. CWWG core regions are underlined and A/T bps in the flanking RRR/YYY regions are in orange color and in bold.

PDB ID	Resolution	R_{free}	DNA sequence		Note	Suppl. Ref.
**Ordered Lys120 side chain**						
2AC0	1.8	0.217	CGGGCATGCCCG			6
3IGK	1.7	0.214	CGGGCATGCCCG			7
3IGL	1.8	0.212	CGGGCATGCCCG			7
4IBU	1.7	0.197	CGGGCATGCCCG		R273C, T284R mutations	8
4IBW	1.8	0.188	CGGGCATGCCCG		R273H, T284R mutations	8
4IBV	2.1	0.229	CGGGCATGCCCG		R273C, S240R mutations	8
3KZ8	1.9	0.266	TGGGCATGCCC	GGGCATGCCC		7
5MCT	1.4	0.189	TGGGCGTGCCC	GGGCGTGCCC		9
5MCU	1.7	0.201	TGGGCBTGCCC	GGGCBTGCCC	B: isoguanine	9
5MCV	1.6	0.188	TGGGCIMGCCC	GGGCIMGCCC	I: Inosine, M: 5mC	9
5MCW	1.9	0.235	TGGGCIMGCCC	GGGCIMGCCC	I: Inosine, M: 5mC	9
5MG7	1.45	0.202	T**A**G**A**CATGCC**T**	GGGCATG**T**C**A**	Pseudo palindromic in asymmetric unit	9
6FJ5	2.05	0.244	T**A**GGCATGCC**T**	**A**GGCATGCC**T**		9
2ADY	2.5	0.251	CGG**A**CATG**T**CCG			6
**Disordered Lys120 side chain**						
3Q05	2.4	0.267	ACGGGCATG**T**C**T**	GGGCATG**T**C**T**CAAA		4
3Q06	3.2	0.314	ACGGGCATG**T**C**T**	GGGCATG**T**C**T**CAAA		4
3TS8	2.8	0.283	AGG**AA**CATG**T**CC	C**AA**CATG**TT**GAGAA		10
4HJE	1.9	0.228	**A**GGCTTG**T**C**T**	**TAA**CTTG**T**G**A**		11
4MZR	2.9	0.281	ACGGGCAAG**T**C**T**	GGGCAAG**T**C**T**CAAA		12
5MF7	1.6	0.233	C**A**GCATG**T**C**T**	**AAA**CATGC**T**CA		9
5BUA	1.8	0.213	GG**A**CATG**T**CC		K120Ac	13
5LGY	2.9	0.277	**A**GGCTTG**T**C**T**	**TAA**CTTG**T**G**A**	K120Ac	13
2GEQ	2.3	0.238	GCGTG**A**GCATGC**T**CAC		Mouse p53	14
3EXJ	2.0	0.26	TTG**A**GCATGC**T**C		Mouse p53	15
3EXL	2.2	0.253	TTG**A**GCATGC**T**C		Mouse p53	15
**Both cases**						
2AHI	1.85	0.225	CGG**A**CATG**T**CCG			6
2ATA	2.2	0.215	**AA**GGCATGCC**TT**			6
3KMD	2.15	0.239	GGGCATGCC**T**	**A**GGCATGCC		16

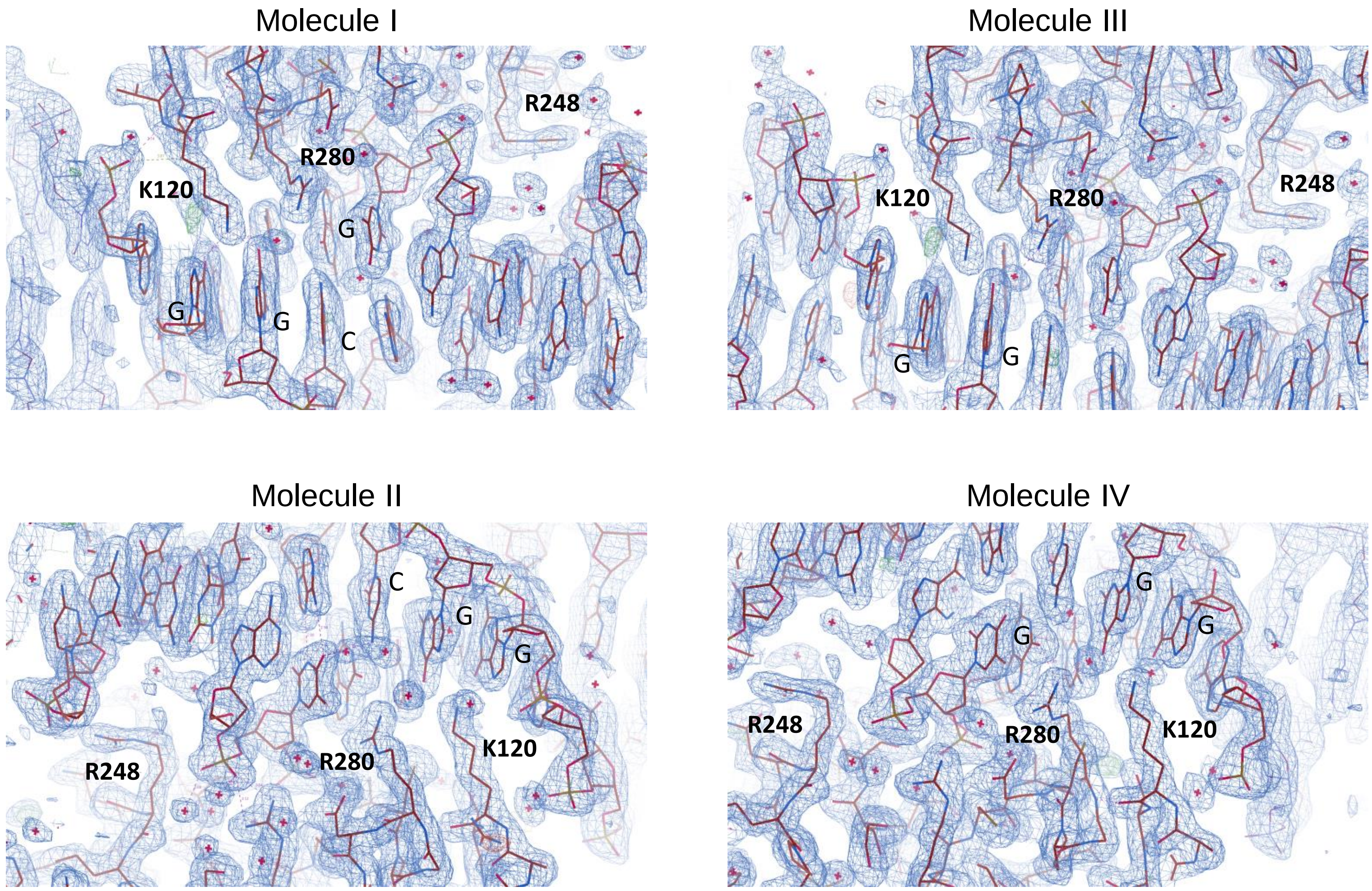

Supplementary Fig. 5: Detailed view of Lys120, Arg248, and Arg280 interactions with a low-affinity, K120R-unbound RE in the individual p53 protomers (PDB: 6FJ5[9]), related to Fig. 3. The $2F_o$-F_c electron density contoured at 1σ above the mean (blue) and the F_o-F_c electron density map contoured at 4σ (green) and -4σ (red) above the mean are shown for the four protomers of the p53 tetramer.

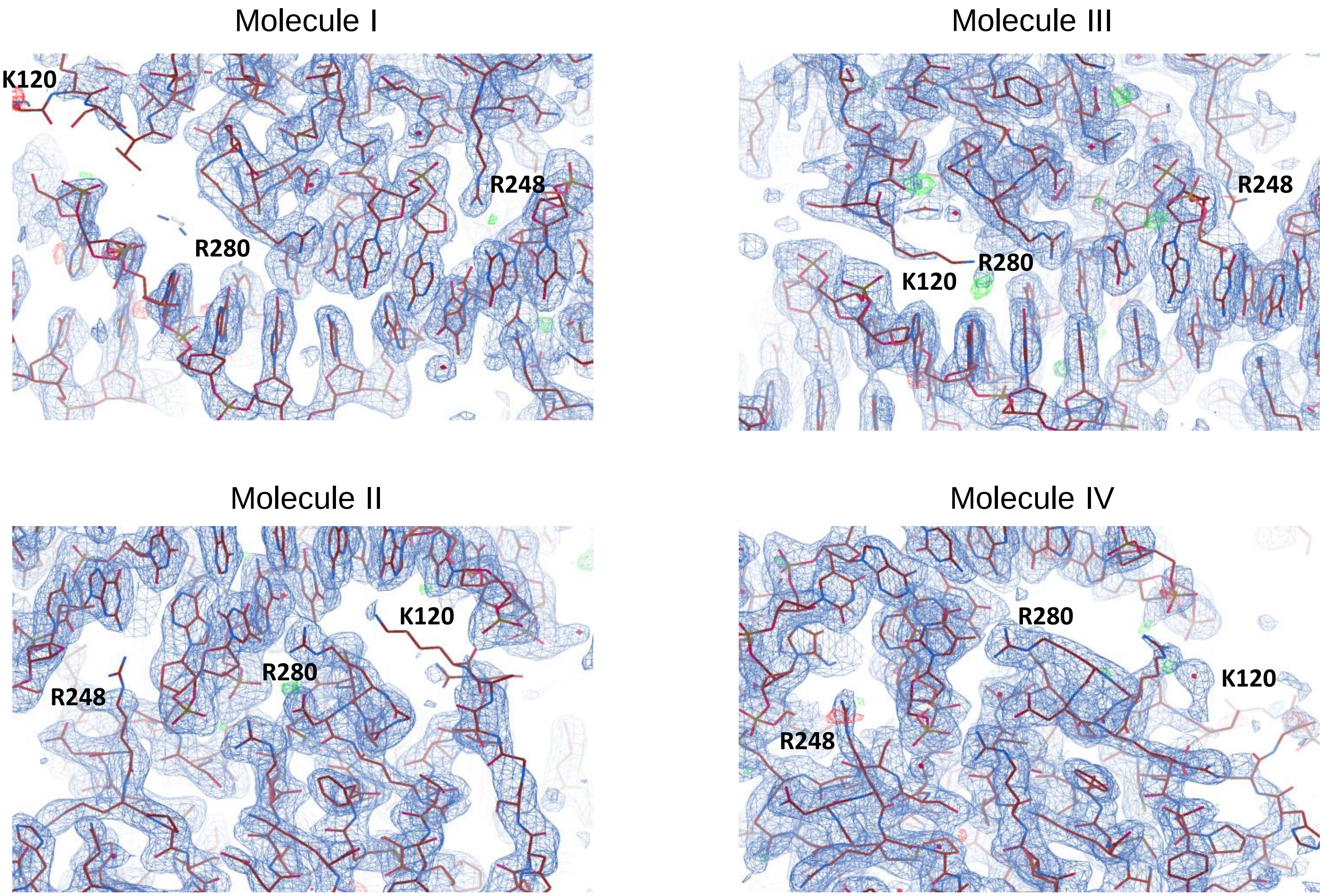

Supplementary Fig. 6: Detailed view of Lys120, Arg248, and Arg280 interactions with a high-affinity, K120R-bound RE in the individual p53 protomers (PDB: 3Q05[4]), related to Fig. 3. The $2F_o$-F_c electron density map contoured at 1σ above the mean (blue) and the F_o-F_c electron density map contoured at 4σ (green) and -4 σ (red) above the mean are shown for the four protomers of the p53 tetramer.

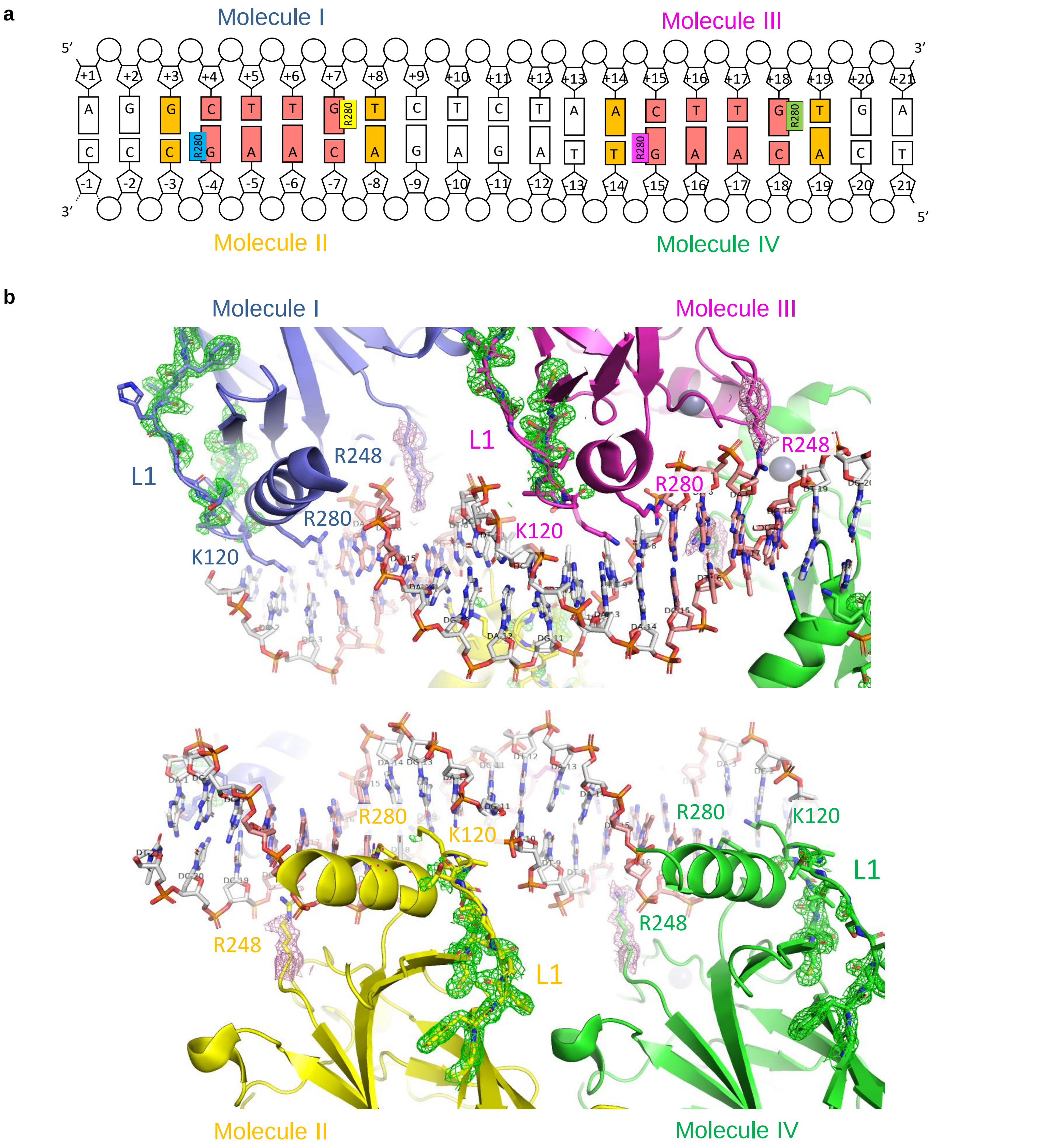

Supplementary Fig. 7: The p53 DNA-binding mode is not affected by the base composition of the CWWG core motif, related to Fig. 3. a, Schematic representation of tetrameric p53 in complex with a RE featuring the core motifs CTTG and CAAG. The four Arg280 residues of tetrameric p53 (molecules I to IV) that specifically interact with guanine of the CTTG and CAAG core regions are marked in blue, yellow, magenta, and green. The CWWG core motif is colored in red, and bases adjacent to the core motif sequences are colored in orange. **b**, Structure of tetrameric p53 in complex with the RE shown in **a** (PDB: 4HJE[11]). The 2Fo-Fc electron density map contoured at 1σ above the mean is shown for the L1 loops in green and for the Arg248 residues in magenta. The side chains of Lys120, Arg248, and Arg280 are also shown. The binding mode for high-affinity REs is adopted based on the prevalent A/T content at positions 3, 8, 13, and 18 and not determined by the dinucleotide core of the CWWG motif.

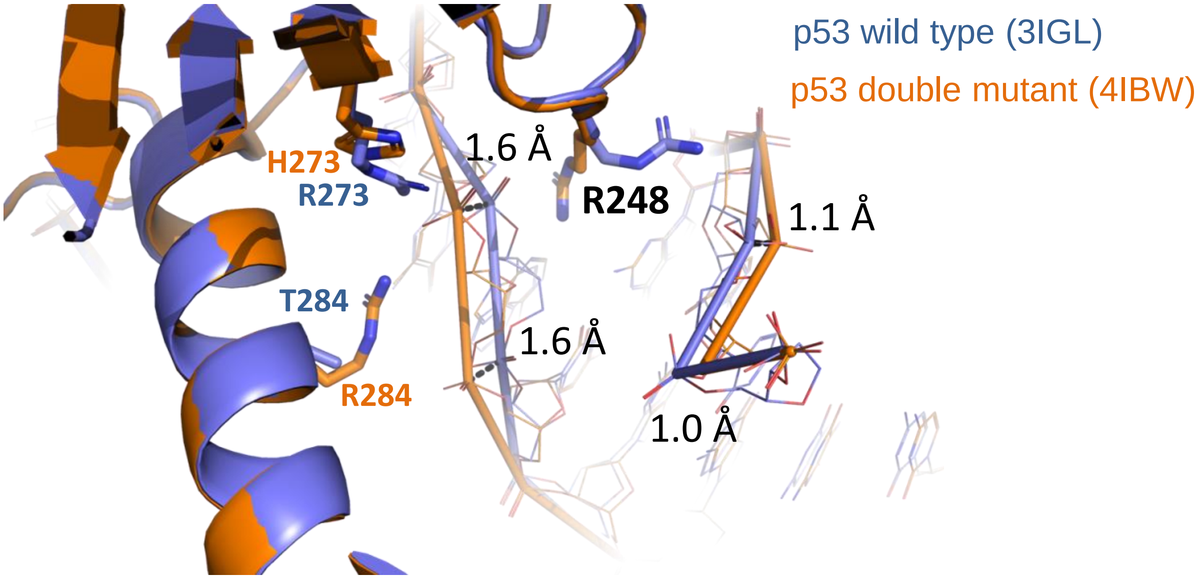

Supplementary Fig. 8: Changing the minor groove causes a switch in Arg248 conformation, related to Fig. 3. A double mutation in p53 (R273H / T284R) widens the minor groove in the region contacting Arg248, as seen in the mutant structure (orange, PDB: 4IBW)[8] with respect to the wild-type structure (dark blue, PDB: 3IGL)[7]. This change causes a switch in Arg248 side-chain conformation, confirming our observation that the Arg248 side-chain conformation is affected by the minor groove width.

a

Name	RE sequence	K_d [nM]			
		WT		K120R	
		200 mM NaCl	275 mM NaCl	200 mM NaCl	275 mM NaCl
NOXA	GAGCgTGTCCGGGCAgGTCg	45	446	68	~2900
NOXA_3/13	GAACgTGTCCGGACAgGTCg	23	60	14	181

NOXA (**blue**) vs. NOXA_3/13 (**red**)

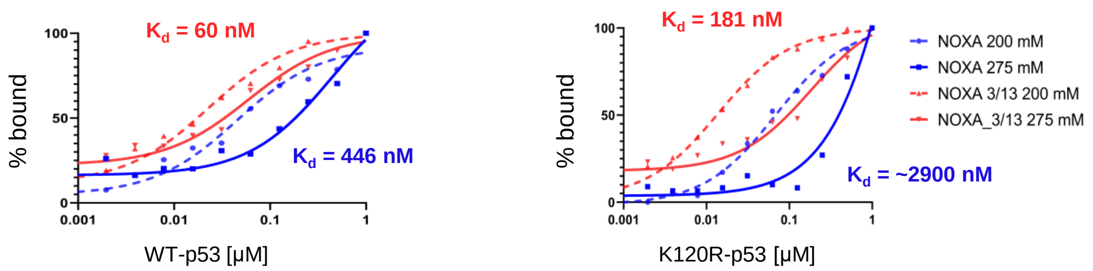

b

Name	RE sequence	K_d [nM]			
		WT		K120R	
		200 mM NaCl	275 mM NaCl	200 mM NaCl	275 mM NaCl
p21	GAACATGTCCcAACATGTTg	26	23	35	43
p21_8/13	GAACATGCCCcAGCATGTTg	33	99	55	479

p21 (**blue**) vs. p21_8/13 (**red**)

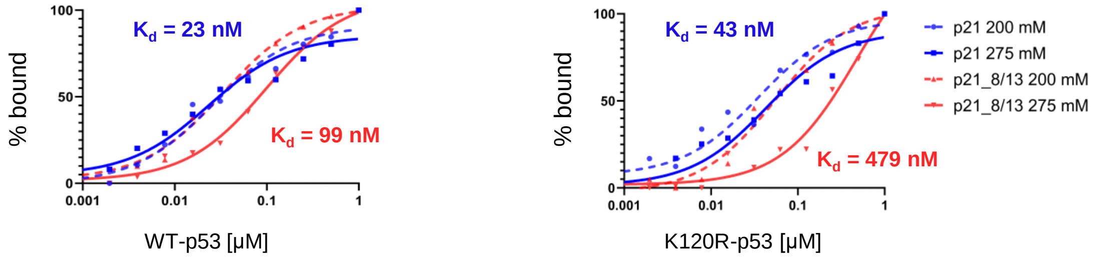

c

Name	RE sequence	K_d [nM]			
		WT		K120R	
		200 mM NaCl	275 mM NaCl	200 mM NaCl	275 mM NaCl
6FJ5	AGGCATGCCTAGGCATGCCT	8	783	140	800
6FJ5_8/18 I/C	AGGCATG(I-)CCTAGICATG(I-)CCT	8	58	72	691

6FJ5 (**blue**) vs. 6FJ5_8/18 I/C (**red**)

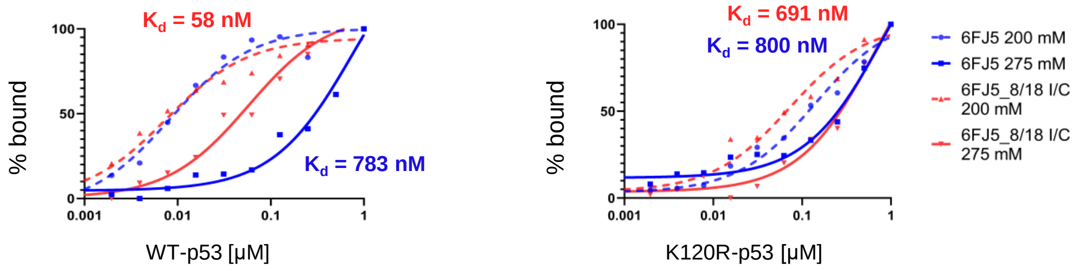

Supplementary Fig. 9: Additional *in vitro* testing of a model on low-affinity/K120R-unbound NOXA RE and 6FJ5 RE, and high-affinity/K120R-bound p21 RE, related to Fig. 4. a, G/C to A/T conversions at positions 3 and 13 in the NOXA RE decrease ~8-fold measured K_d values for WT p53 at high salt concentrations (from 446 nM to 60 nM), and restore K120R-p53 binding. **b**, A/T to G/C conversions at positions 8 and 13 in the p21 RE increase measured K_d values for WT-p53 (from 23 nM to 99 nM) and K120R-p53 (form 43 nM to 479 nM) **c,** G/C to I/C conversions at positions 8 and 18 in the 6FJ5 RE increase the affinity of this RE for the WT p53, as evident in ~12-fold lower K_d values (from 783 nM to 58 nM), but do not have an impact on K120R-p53 binding. Data are presented as in Fig. 4a and b. Calculated K_d values (mean of two independent measurements) are reported on graphs and in tables. Lower case letters in RE sequences indicate mismatches from p53 RE consensus. Salmon-colored letters indicate converted nucleotides. I (inosine) and C (cytosine) in salmon color indicate I/C bps. Letters in parenthesis indicate a nucleotide of the I-C bp found on the complementary strand. Source data are provided as a Source Data file.

a

Gene	Sequence
APBB2	AGGCATGTCCCAACATGCCC
C8orf34	AGGCATGCCCCAACATGCCC
SSUH2	AGGCATGCCCCAGCATGCCC

b

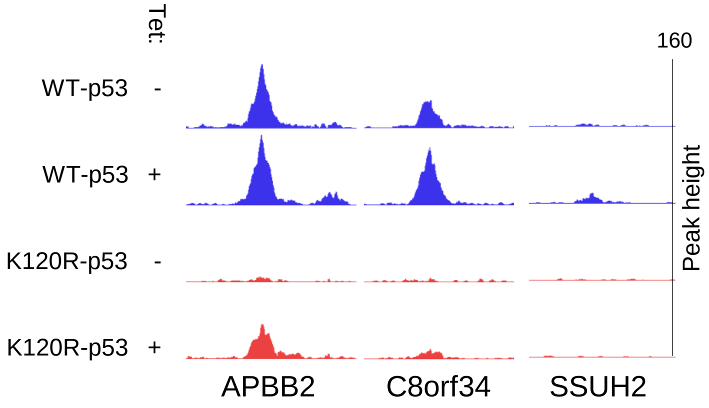

Supplementary Fig. 10: Natural response elements with a single G/C vs. A/T difference at positions 8 and/or 13, and their impact on a WT and K120R-p53 occupancy *in vivo*, related to Fig. 5. a, Sequences of previously identified[10] REs at *APBB2*, *C8orf34*, and *SSUH2* genes (all with an unknown role in p53 pathways) with colored positions 3, 8, 13, and 18 (A/T in blue and G/C in salmon). **b,** UCSC Genome Browser Tracks screenshots of WT p53 (blue) and K120R-p53 (salmon) binding on corresponding REs. A single nucleotide substitution from A/T (in a RE linked to *APBB2* gene) to G/C (in REs linked to *c8orf34* gene) at position 8 slightly decreases WT p53 occupancy and negatively impacts K120R-p53 binding. An additional substitution from A/T to G/C at the position 13 (in a RE linked to *SSUSH2* gene), which makes this RE having all four G/C bps at positions 3, 8, 13, and 18, completely diminishes both WT and K120R-p53 binding.

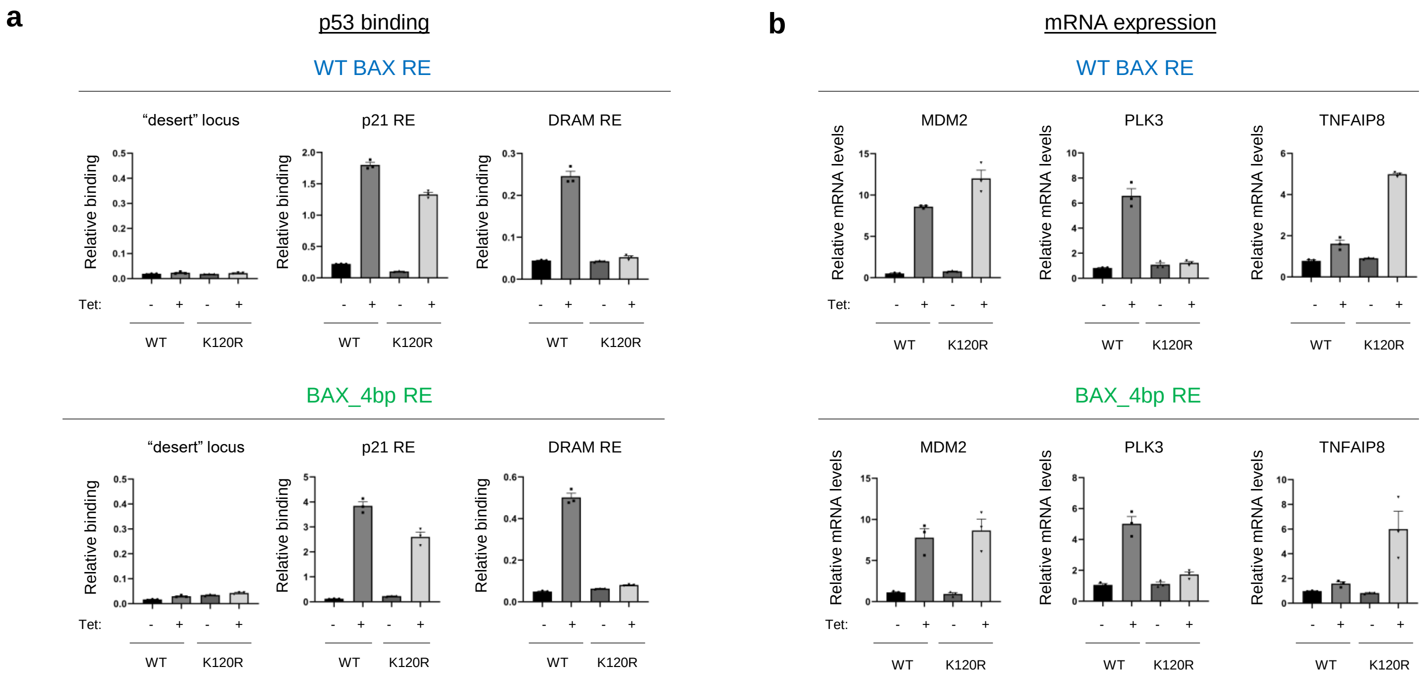

Supplementary Fig. 11: Genome editing of the BAX RE does not impact p53 binding to other natural REs or expression of other p53-dependent target genes, related to Fig. 5. a, ChIP signals at two natural p53 REs (p21 RE and DRAM RE) or "desert" site (locus on the chromosome 12, void of any regulatory or coding elements, used as a negative control) for WT and K120R-p53 proteins in cells with the wild-type BAX RE (top) or CRISPR/Cas9-edited BAX_4bp RE (bottom). Graphs represent precipitated DNA relative to an input (total) DNA and show cumulative data from three independent experiments (mean ± s.e.m.). No change in WT or K120R-p53 binding could be observed at any analyzed site between edited and non-edited cell lines. **b**, Quantitative polymerase chain reaction (qPCR) of *MDM2*, *PLK3*, and *TNFAIP8* mRNA (represented relative to *GAPDH* mRNA) from the same cells as in panel **a**. The graphs show cumulative data from three independent experiments (mean ± s.e.m.). Similar to panel **a**, no change in mRNA expression patterns could be observed. Source data are provided as a Source Data file.

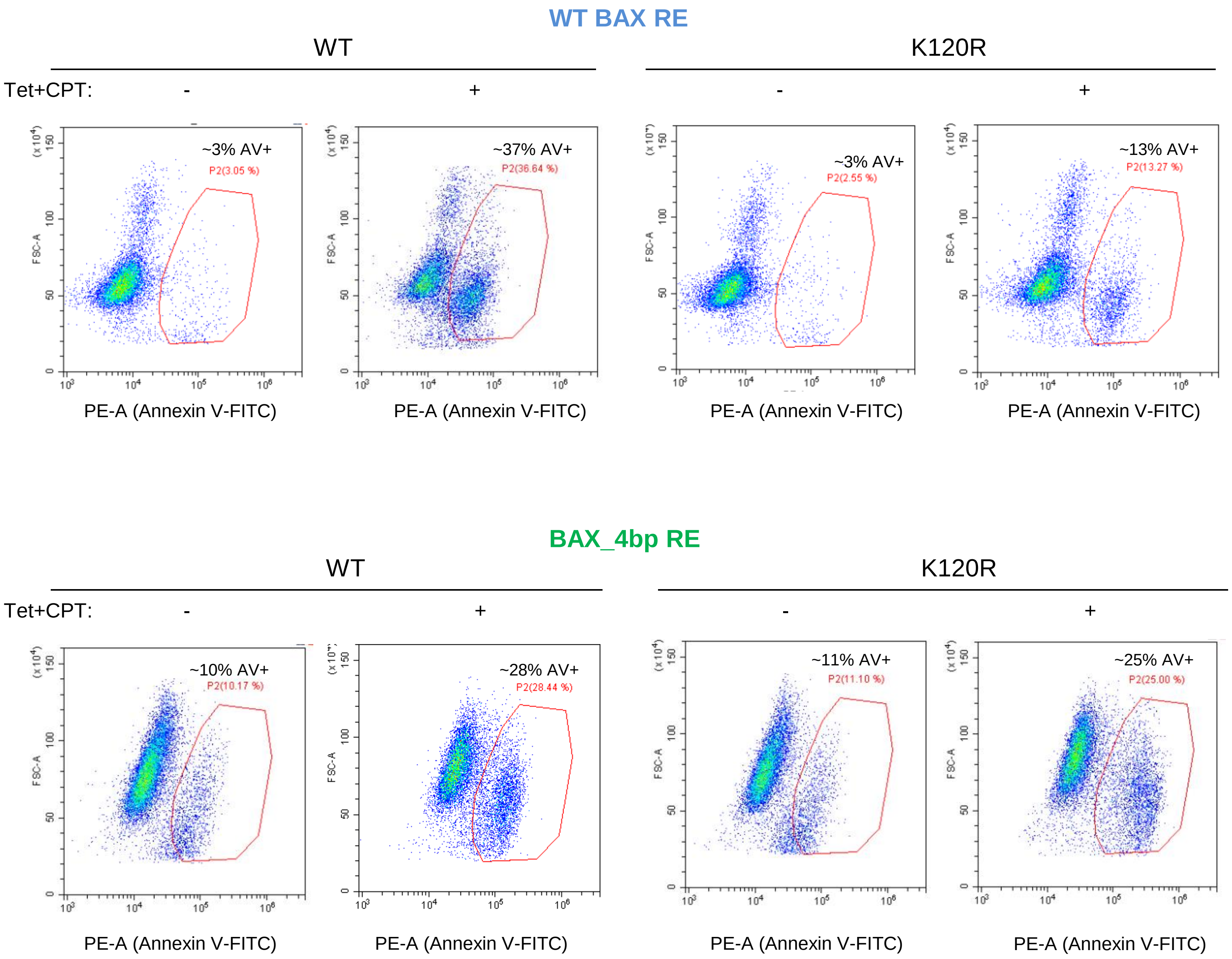

Supplementary Fig. 12: The original source images for a representative flow cytometry data, related to Fig. 5. Red polygons depict Annexin V gating strategy. Percentages of Annexin V positive cells (mean ± s.e.m.) from three independent experiments are depicted on graphs in Fig. 5e. Data was collected using the CytExpert v1.2 (Beckman Coulter) software. Each analysis was performed in a population of 10,000 cells. AV+, Annexin V positive.

Supplementary Table 2: Antibodies used in this paper.

Target antigen	Target species	Host species/clone	Dilution	Manufacturer
p53	Human	Mouse/monoclonal DO-1	1:1000	Santa Cruz Bio.
Gapdh	Human	Rabbit/monoclonal D16H11	1:2000	Cell Signaling
Tet Repressor	E. coli	Rabbit polyclonal	1:2000	Milipore Sigma
Bax	Human	Rabbit/monoclonal EPR18284	1:1000	Abcam
Mdm2	Human	Rabbit polyclonal	1:1000	Oncogene Research
Mouse IgG	Mouse	Goat polyclonal	1:20000	Milipore Sigma
Rabbit IgG	Rabbit	Goat polyclonal	1:20000	Milipore Sigma

Supplementary Table 3: cDNA primers and corresponding sequences used for qRT-PCR.

Gene (cDNA) primer	Forward (5' to 3')	Reverse (5' to 3')
GAPDH	TGGGCTACACTGAGCACCAG	GGGTGTCGCTGTTGAAGTCA
BAX	TCCCCCCGAGAGGTCTTTT	CGGCCCCAGTTGAAGTTG
MDM2	TGTTTGGCGTGCCAAGCTTCTC	CACAGATGTACCTGAGTCCGATG
PLK3	CCCCTCCTTGTGACTTTTGTG	GAAGGTGGGAAGCGAGGTAAG
TNFAIP8	GGCCGTTCAGGCACAAAA	AGGTGGTGCTGGCGATGGATTT

Supplementary Table 4: ChIP primers and corresponding sequences used in ChIP-PCR. BS (binding site); RE (response element).

ChIP primer	Forward (5' to 3')	Reverse (5' to 3')
Chr12 "desert" BS	CAATATCCAGCGAAAAGGAAGCT	GGGATGATGTGTGGGTTTTACC
BAX RE	AGGCTGAGACGGGGTTATCT	GCGCAGAAGGAATTAGCAAG
p21 RE	GGGTTTCTGGCCATCAGGA	CCACCAGCCTCTTCTATGGC
DRAM RE	CAAGCTCCCCGTGCAAGT	CACCCTACAAGTGCTTACACCAGAT

Supplementary Table 5: Sequences of fluorescently labelled 36-bp DNA oligos used in FP assays, with an actual RE sequences (as seen in Fig. 2a) in bold. 6-FAM (6-carboxyfluorescein); pdb (protein data bank); I/C (inosine/cytosine); FP (fluorescent polarization).

Oligo/RE name	Forward (5' to 3')
p21	6-FAM-ATGATGAT**GAACATGTCCcAACATGTTg**ATATGATG
MDM2	6-FAM-ATGATGAT**AGACAAGTCaGGACTTaaCT**ATATGATG
APBB2	6-FAM-ATGATGAT**AGGCATGTCCcAACATGCCC**ATATGATG
3Q05 pdb	6-FAM-ATGATGA**TGGGCATGTCTGGGCATGTCT**ATATGATG
"Pro-apoptotic"	6-FAM-ATGATGAT**GGGCATGCCCGGGCATGCCC**ATATGATG
BAX	6-FAM-ATGATGAT**GGGCAgGCCCGGGCTTGTCg**ATATGATG
NOXA	6-FAM-ATGATGAT**GAGCgTGTCCGGGCAgGTCg**ATATGATG
6FJ5 pdb	6-FAM-ATGATGAT**AGGCATGCCTAGGCATGCCT**ATATGATG
BAX_8/13	6-FAM-ATGATGAT**GGGCAgGTCCGGACTTGTCg**ATATGATG
NOXA_3/13	6-FAM-ATGATGAT**GAACGTGTCCGGACAGGTCG**ATATGATG
p21_8/13	6-FAM-ATGATGAT**GAACATGCCCcAGCATGTTg**ATATGATG
BAX_8/13 I/C	6-FAM-ATGATGAT**GGGCAgG(I-)CCCGGICTTGTCg**ATATGATG
6FJ5_8/18 I/C	6-FAM-ATGATGAT**AGGCATG(I-)CCTAGICATG(I-)CCT**ATATGATG

Supplementary Table 6: DNA oligos used for the creation of genetically edited BAX RE ("BAX_4bp") by CRISPR/Cas9. HDR (homology directed repair); PCR (polymerase chain reaction); Fwd (forward); Rev (reverse).

Oligo	Sequence (5' to 3')
HDR template	GAACGCTATATGCATCCAGACCCCTGGCCTGAGGGGGCAGAGGCGAGACCCGC CCCTCCCACCAGTGCCAGAGGCAGGAAGTGGTGCGGGCGACAAGTTGGGACCT GCCCGGGGGCTTGGCTTCCGAGATCCCCTGGTTCTCGAGGGTCCGAGAGCGCC AGAGAGGCAGGGATCGGGACACGGGGATCAGAGAGCCAGGA
PCR primers used for a clonal identification	Fwd: GCACTGAGGGGCAGAAACTC<br>Rev: CAGAATCAGAACGGGGTGTCAC

Supplementary References

1. Fischer, M. Census and evaluation of p53 target genes. *Oncogene* **36**, 3943–3956 (2017).
2. Kuribayashi, K., Finnberg, N. K., Jeffers, J. R., Zambetti, G. P. & El-Deiry, W. S. The relative contribution of pro-apoptotic p53-target genes in the triggering of apoptosis following DNA damage in vitro and in vivo. *Cell Cycle* **10**, 2380–2389 (2011).
3. Le Pen, J. *et al.* Constitutive p53 heightens mitochondrial apoptotic priming and favors cell death induction by BH3 mimetic inhibitors of BCL-xL. *Cell Death Dis* **7**, e2083–e2083 (2016).
4. Petty, T. J. *et al.* An induced fit mechanism regulates p53 DNA binding kinetics to confer sequence specificity: p53 conformational switch. *The EMBO Journal* **30**, 2167–2176 (2011).
5. Emamzadah, S., Tropia, L., Vincenti, I., Falquet, B. & Halazonetis, T. D. Reversal of the DNA-Binding-Induced Loop L1 Conformational Switch in an Engineered Human p53 Protein. *Journal of Molecular Biology* **426**, 936–944 (2014).
6. Kitayner, M. *et al.* Structural Basis of DNA Recognition by p53 Tetramers. *Molecular Cell* **22**, 741–753 (2006).
7. Kitayner, M. *et al.* Diversity in DNA recognition by p53 revealed by crystal structures with Hoogsteen base pairs. *Nat Struct Mol Biol* **17**, 423–429 (2010).
8. Eldar, A., Rozenberg, H., Diskin-Posner, Y., Rohs, R. & Shakked, Z. Structural studies of p53 inactivation by DNA-contact mutations and its rescue by suppressor mutations via alternative protein-DNA interactions. *Nucleic Acids Research* **41**, 8748–8759 (2013).
9. Golovenko, D. *et al.* New Insights into the Role of DNA Shape on Its Recognition by p53 Proteins. *Structure* **26**, 1237-1250.e6 (2018).

10. Emamzadah, S., Tropia, L. & Halazonetis, T. D. Crystal Structure of a Multidomain Human p53 Tetramer Bound to the Natural CDKN1A (p21) p53-Response Element. *Molecular Cancer Research* **9**, 1493–1499 (2011).

11. Chen, Y. *et al.* Structure of p53 binding to the BAX response element reveals DNA unwinding and compression to accommodate base-pair insertion. *Nucleic Acids Research* **41**, 8368–8376 (2013).

12. Emamzadah, S., Tropia, L., Vincenti, I., Falquet, B. & Halazonetis, T. D. Reversal of the DNA-Binding-Induced Loop L1 Conformational Switch in an Engineered Human p53 Protein. *Journal of Molecular Biology* **426**, 936–944 (2014).

13. Vainer, R., Cohen, S., Shahar, A., Zarivach, R. & Arbely, E. Structural Basis for p53 Lys120-Acetylation-Dependent DNA-Binding Mode. *Journal of Molecular Biology* **428**, 3013–3025 (2016).

14. Ho, W. C., Fitzgerald, M. X. & Marmorstein, R. Structure of the p53 Core Domain Dimer Bound to DNA. *J. Biol. Chem.* **281**, 20494–20502 (2006).

15. Malecka, K. A., Ho, W. C. & Marmorstein, R. Crystal structure of a p53 core tetramer bound to DNA. *Oncogene* **28**, 325–333 (2009).

16. Chen, Y., Dey, R. & Chen, L. Crystal Structure of the p53 Core Domain Bound to a Full Consensus Site as a Self-Assembled Tetramer. *Structure* **18**, 246–256 (2010).
