# Supplementary material for: Distinct mechanisms control genome recognition by p53 at its target genes linked to different cell fates
Source: Nat Commun. 2021 Jan 20;12:484. doi: 10.1038/s41467-020-20783-z (PMC7817693; doi:10.1038/s41467-020-20783-z)
Supplement: Supplementary file 2 — Reporting Summary [file 41467_2020_20783_MOESM2_ESM.pdf]

nature research

Corresponding author(s): Steven B. McMahon

Last updated by author(s): Dec 13, 2020

Reporting Summary

Nature Research wishes to improve the reproducibility of the work that we publish. This form provides structure for consistency and transparency in reporting. For further information on Nature Research policies, see our Editorial Policies and the Editorial Policy Checklist.

Statistics

For all statistical analyses, confirm that the following items are present in the figure legend, table legend, main text, or Methods section.

n/a	Confirmed	
☐	☒	The exact sample size (*n*) for each experimental group/condition, given as a discrete number and unit of measurement
☐	☒	A statement on whether measurements were taken from distinct samples or whether the same sample was measured repeatedly
☐	☒	The statistical test(s) used AND whether they are one- or two-sided<br>*Only common tests should be described solely by name; describe more complex techniques in the Methods section.*
☒	☐	A description of all covariates tested
☒	☐	A description of any assumptions or corrections, such as tests of normality and adjustment for multiple comparisons
☐	☒	A full description of the statistical parameters including central tendency (e.g. means) or other basic estimates (e.g. regression coefficient) AND variation (e.g. standard deviation) or associated estimates of uncertainty (e.g. confidence intervals)
☐	☒	For null hypothesis testing, the test statistic (e.g. *F*, *t*, *r*) with confidence intervals, effect sizes, degrees of freedom and *P* value noted<br>*Give P values as exact values whenever suitable.*
☒	☐	For Bayesian analysis, information on the choice of priors and Markov chain Monte Carlo settings
☒	☐	For hierarchical and complex designs, identification of the appropriate level for tests and full reporting of outcomes
☒	☐	Estimates of effect sizes (e.g. Cohen's *d*, Pearson's *r*), indicating how they were calculated

Our web collection on statistics for biologists contains articles on many of the points above.

Software and code

Policy information about availability of computer code

Data collection	For quantification of cDNA/DNA: Step One Plus Real Time-PCR System v2.3 (Applied Biosystems);<br>For library sequencing: NextSeq 500 Sequencing System (Illumina);<br>Fluorescence polarization experiments were carried out on a: VICTOR3 (Perkin Elmer);<br>For luciferase reporter assays: Infinite 200 Pro v3.37 (Tecan);<br>Flow cytometry analysis were performed by: CytExpert v1.2 (Beckman Coulter)<br>Microscopic images were generated using: ZEN v2.3 (Zeiss)
Data analysis	For DNA library insert size analysis: BioAnalyzer (Agilent);<br>For ChIP-seq reads alignment: BWA-MEM v0.7.12;<br>For ChIP-seq peak calling: MACS2;<br>For ChIP-seq peak visualization: HOMER v4.10;<br>For creation of DNA logos: WebLogo v3;<br>For Kd measurements (binding curve fitting): GraphPad Prism v8.4.2;<br>For omit maps creation: PHENIX v1.6;<br>For DNA groove width analysis: 3DNA v2;<br>Molecular graphics were generated using : PyMol v2.3;<br>For statistical analysis: GraphPad Prism v8.4.2; Microsoft Excel 2010.

For manuscripts utilizing custom algorithms or software that are central to the research but not yet described in published literature, software must be made available to editors and reviewers. We strongly encourage code deposition in a community repository (e.g. GitHub). See the Nature Research guidelines for submitting code & software for further information.

Data

Policy information about availability of data

All manuscripts must include a data availability statement. This statement should provide the following information, where applicable:

- Accession codes, unique identifiers, or web links for publicly available datasets
- A list of figures that have associated raw data
- A description of any restrictions on data availability

The data supporting the study are available within the paper and its Supplementary Information files. ChIP-sequencing data generated during the study have been deposited in the Gene Expression Omnibus (GEO) repository and are available under GEO accession number GSE159945 (https://www.ncbi.nlm.nih.gov/geo/query/acc.cgi?acc=GSE159945). UCSC Genome Browser session for the visualization of generated ChIP-sequencing data is available from the corresponding author on request. Structural figures of p53 in a complex with different DNA were generated from publicly available datasets from the Protein Data Bank (3Q05 [DOI: 10.2210/pdb3q05/pdb]; 6FJ5 [DOI: 10.2210/pdb6FJ5/pdb]; 4HJE [DOI: 10.2210/pdb4HJE/pdb]; 4IBW [DOI: 10.2210/pdb4IBW/pdb]; 3IGL [DOI: 10.2210/pdb3IGL/pdb].
Source data for Figures 1 - 5, Table 1, and Supplementary Data figures are provided with this paper as the Source Data file.

Field-specific reporting

Please select the one below that is the best fit for your research. If you are not sure, read the appropriate sections before making your selection.

☒ Life sciences ☐ Behavioural & social sciences ☐ Ecological, evolutionary & environmental sciences

For a reference copy of the document with all sections, see nature.com/documents/nr-reporting-summary-flat.pdf

Life sciences study design

All studies must disclose on these points even when the disclosure is negative.

Sample size	The in vivo data presented here are almost entirely based on cultured human cells. All experiments on cultured cells (WB, RNA expression, Luciferase assays, Annexin V staining, Cas3/7 staining and imaging, etc.) were performed three or more times (as indicated in the text), to allow tests of significance using the Student's t-test.<br>For in vitro data, sample sizes were chosen according to the basis of previous publications (Hashimoto et. al, 2014, ref. 20), common practice and preliminary experiments.
Data exclusions	No data were excluded from the analysis in this study.
Replication	All experiments were repeated at least twice (in vitro) and the vast majority of in vivo experiments were repeated 3-6 times to assure reproducibility. All attempts of replication were successful.
Randomization	H1299 cells of different genotypes (WT-p53, K120R-p53, etc.) were cultured separately and were randomly treated with the various stimuli, for different time point, followed by a different assay analysis (SDS-PAGE, protein or RNA purification, etc.). All cells of the same population (same genotype) were randomly seeded from the identical cell population (same plate) into experimental groups prior the treatment, to avoid any bias.
Blinding	No blinding was used in this study.<br>The analyses of cultured cells in this study (which were used in majority of experiments) involved multiple conditions, genes or proteins that required multiple steps to process, including cell culturing, plasmid transfections, protein and DNA purifications, analyses by SDS-PAGE, flow cytometry, microscopy, etc. A single scientist performed each of these multiple steps and it would be difficult to conduct blind study under these conditions.<br>Most of in vitro experiments and all structural analyses were performed using computational algorithms and softwares, so scientists were not blinded.

Reporting for specific materials, systems and methods

We require information from authors about some types of materials, experimental systems and methods used in many studies. Here, indicate whether each material, system or method listed is relevant to your study. If you are not sure if a list item applies to your research, read the appropriate section before selecting a response.

Materials & experimental systems

n/a	Involved in the study
☐	☒ Antibodies
☐	☒ Eukaryotic cell lines
☒	☐ Palaeontology and archaeology
☒	☐ Animals and other organisms
☒	☐ Human research participants
☒	☐ Clinical data
☒	☐ Dual use research of concern

Methods

n/a	Involved in the study
☐	☒ ChIP-seq
☐	☒ Flow cytometry
☒	☐ MRI-based neuroimaging

Antibodies

Antibodies used

1. monoclonal mouse anti-p53 (DO-1), Cat. No. sc-126, Santa Cruz Biotechnology, Dilution 1:1000, Lot# D0915
2. monoclonal rabbit anti GAPDH (D16H11), Cat. No. 5174S, Cell Signaling, Dilution 1:2000, Lot# 8
3. polyclonal rabbit anti Tet Repressor, Cat. No. T0951, Milipore Sigma, Dilution 1:1000, Lot# SLBD2542V
4. monoclonal rabbit anti BAX (EPR18284), Cat. No. ab182734, Abcam, Dilution 1:1000, Lot# N/A
5. monoclonal mouse anti MDM2 (Ab-1), Cat. No. OP46, Oncogene Research, Dilution 1:1000, Lot# D11888-1
6. polyclonal goat anti mouse IgG, Cat. No. AP124P, Milipore Sigma, Dilution 1:20,000, Lot# 2966512
7. polyclonal goat anti rabbit IgG, Cat. No. AP132P, Milipore Sigma, Dilution 1:20,000, Lot# 2946002
8. polyclonal rabbit anti p53 (FL393-x), Cat. No. sc-6243x, Santa Cruz Biotechnology, Dilution 1:50, Lot# D2015

Validation

1. anti-p53:
Gerin I, et al. Phosphoglycolate has profound metabolic effects but most likely no role in a metabolic DNA response in cancer cell lines. Biochem J. 476(4):629-643 (2019); https://www.ncbi.nlm.nih.gov/pmc/articles/PMC6380167/
Sun M, et al. Targeting the Chromosomal Passenger Complex Subunit INCENP Induces Polyploidization, Apoptosis, and Senescence in Neuroblastoma. Cancer Res. 79(19):4937-4950 (2019); https://www.ncbi.nlm.nih.gov/pmc/articles/PMC6774870/

2. anti-GAPDH:
Hennighausen and Lee. Activation of the SARS-CoV-2 Receptor Ace2 through JAK/STAT-Dependent Enhancers during Pregnancy. Cell Rep. 32(13):108199 (2020); https://pubmed.ncbi.nlm.nih.gov/32966801/
Yang B, et. al. Panax notoginseng saponins alleviates advanced glycation end product-induced apoptosis by upregulating SIRT1 and antioxidant expression levels in HUVECs. Exp. Ther. Med. 20(5):99 (2020); https://www.ncbi.nlm.nih.gov/pmc/articles/PMC7506886/

3. anti-Tet Repressor:
Zhang X, et al. FOXO1 is an essential regulator of pluripotency in human embryonic stem cells. Nature Cell Biology 13(9) 1092-1092, (2011); https://pubmed.ncbi.nlm.nih.gov/21804543/

4. anti-BAX:
Yang J, et al. T-2 Toxin-Induced Oxidative Stress Leads to Imbalance of Mitochondrial Fission and Fusion to Activate Cellular Apoptosis in the Human Liver 7702 Cell Line. Toxins. 12(1):43 (2020); https://pubmed.ncbi.nlm.nih.gov/31936883/
Mo J, et al. AVE 0991 attenuates oxidative stress and neuronal apoptosis via Mas/PKA/CREB/UCP-2 pathway after subarachnoid hemorrhage in rats. Redox Biol. 20:75-86 (2019); https://pubmed.ncbi.nlm.nih.gov/30296700/

5. anti-MDM2:
Warburton HE, et al. p53 regulation and function in renal cell carcinoma. Cancer Res. 65, 6498-6503 (2005); https://www.ncbi.nlm.nih.gov/pmc/articles/PMC3536467/

8. polyclonal anti-p53:
Lee KY, et al. Loss of fragile X protein FMRP impairs homeostatic synaptic downscaling through tumor suppressor p53 and ubiquitin E3 ligase Nedd4-2. Human molecular genetics 27(16):2805-2816 (2018); https://pubmed.ncbi.nlm.nih.gov/29771335/
Schafer C, et al. Class I histone deacetylases regulate p53/NF-κB crosstalk in cancer cells. Cell Sign. 29:218-225 (2017); https://pubmed.ncbi.nlm.nih.gov/27838375/

Eukaryotic cell lines

Policy information about cell lines

Cell line source(s)
H1299 cells were purchased from ATCC (Cat. No. CRL-5803).

Authentication
Authentication was performed by the vendors (ATCC). Primary stocks were expanded and frozen as aliquots directly upon receipt from ATCC. Low passage number cells were utilized throughout these studies and primary aliquots of cells thawed approximately every two months in order to avoid contamination from other cell lines.

Mycoplasma contamination
Cell lines were regularly tested and verified to be mycoplasma negative using LookOut Mycoplasma PCR Detection Kit (Sigma-Aldrich).

Commonly misidentified lines
(See ICLAC register)
No commonly misidentified cell lines were used.

ChIP-seq

Data deposition

☒ Confirm that both raw and final processed data have been deposited in a public database such as GEO.

☒ Confirm that you have deposited or provided access to graph files (e.g. BED files) for the called peaks.

Data access links<br>*May remain private before publication.*	https://www.ncbi.nlm.nih.gov/geo/query/acc.cgi?acc=GSE159945
Files in database submission	Processed data files:<br>WT_NT_peaks.xls<br>WT_TET_peaks.xls<br>K120R_NT_peaks.xls<br>K120R_TET_peaks.xls<br>WT_NT_summits.bed<br>WT_TET_summits.bed<br>K120R_NT_summits.bed<br>K120R_TET_summits.bed<br>WT_NT_peaks.narrowPeak<br>WT_TET_peaks.narrowPeak<br>K120R_NT_peaks.narrowPeak<br>K120R_TET_peaks.narrowPeak<br><br>Raw files:<br>McMahon_13_WT_NT_p53_IP_11-10-16.unique.bam<br>McMahon_14_WT_TET_p53_IP_11-10-16.unique.bam<br>McMahon_16_K120R_NT_p53_IP_11-10-16.unique.bam<br>McMahon_17_K120R_TET_p53_IP_11-10-16.unique.bam
Genome browser session<br>(e.g. UCSC)	No longer applicable.

Methodology

Replicates	WT (-), WT (+), K120R (-) and K120R (+). (-) samples indicate non treated cells with no p53, and (+) indicates Tetracycline-treated cells that express either WT or K120R-p53.
Sequencing depth	75-base single-end sequencing was performed.<br>WT (-) total reads: 36951130, uniquely mapped reads: 35457575;<br>WT (+) total reads: 37669535, uniquely mapped reads: 35939209;<br>K120R (-) total reads: 38364980, uniquely mapped reads: 36422291;<br>K120R (+) total reads: 38467607, uniquely mapped reads: 37044021
Antibodies	Rabbit polyclonal anti p53 (FL393x), sc-6243, Santa Cruz Biotechnology. All information available on vendor website.
Peak calling parameters	The read mapping command with all default parameters except number of parallel threads -t (bwa):<br>bwa mem -t 6 /data/reference/genomes/hg19/hg19.concatenated.fa *.fastq.gz<br>The peak calling command with all default parameters (MACS2):<br>macs2 callpeak -t <ip file *.unique.bam> -c <input file *.unique.bam>
Data quality	MACS2 peak calling has a default FDR cutoff of 0.05 and only those peaks were analyzed. The number of significantly enriched peaks in each replicate was detected by setting an enrichment cutoff of 0.3 counts per million reads, based on preliminary data and previous findings.
Software	For ChIP-seq reads alignment: BWA-MEM v0.7.12;<br>For ChIP-seq peak calling: MACS2;<br>For ChIP-seq peak visualization: HOMER v4.10.

Flow Cytometry

Plots

Confirm that:

☒ The axis labels state the marker and fluorochrome used (e.g. CD4-FITC).

☒ The axis scales are clearly visible. Include numbers along axes only for bottom left plot of group (a 'group' is an analysis of identical markers).

☒ All plots are contour plots with outliers or pseudocolor plots.

☒ A numerical value for number of cells or percentage (with statistics) is provided.

Methodology

Sample preparation	Described in Methods section ("Annexin V staining").
Instrument	CytoFlex (Beckman Coulter).
Software	For data collection and analysis we used CytExpert v1.2 (Beckman Coulter).
Cell population abundance	Each analysis was performed in a population of 10,000 cells. (as described in Methods section)
Gating strategy	For each analysis preliminary FSC/SSC gates were approximately: > 1 x 10^5 (below that what considered cell debris), and < 10 x 10^5.<br>In general, Annexin V positive cells exhibited PE-A channel staining value > 4*10^4. "Negative" cells had a value of approximately 10^3 - 3*10^4.

☒ Tick this box to confirm that a figure exemplifying the gating strategy is provided in the Supplementary Information.
